# Fertility restorer gene *CaRf* and PepperSNP50K provide a promising breeding system for hybrid pepper

**DOI:** 10.1093/hr/uhae223

**Published:** 2024-10-01

**Authors:** Bingqian Tang, Huiping Yang, Qinbiao Yin, Wu Miao, Yuting Lei, Qingzhi Cui, Jiawen Cheng, Xinhao Zhang, Ying Chen, Juan Du, Lingling Xie, Shunxue Tang, Meiqi Wang, Jiayue Li, Mingyue Cao, Li Chen, Fangling Xie, Xiumin Li, Fan Zhu, Zhongyi Wang, Cheng Xiong, Xiongze Dai, Xuexiao Zou, Feng Liu

**Affiliations:** Engineering Research Center of Education, Ministry for Germplasm Innovation and Breeding New Varieties of Horticultural Crops, Key Laboratory for Vegetable Biology of Hunan Province, College of Horticulture, Hunan Agricultural University, Changsha 410128, China; Engineering Research Center of Education, Ministry for Germplasm Innovation and Breeding New Varieties of Horticultural Crops, Key Laboratory for Vegetable Biology of Hunan Province, College of Horticulture, Hunan Agricultural University, Changsha 410128, China; Engineering Research Center of Education, Ministry for Germplasm Innovation and Breeding New Varieties of Horticultural Crops, Key Laboratory for Vegetable Biology of Hunan Province, College of Horticulture, Hunan Agricultural University, Changsha 410128, China; Hunan Xiangyan Seed Industry Co., Ltd, Changsha 410125, China; Higentec Co. Ltd., Changsha, Hunan, 410125, China; Engineering Research Center of Education, Ministry for Germplasm Innovation and Breeding New Varieties of Horticultural Crops, Key Laboratory for Vegetable Biology of Hunan Province, College of Horticulture, Hunan Agricultural University, Changsha 410128, China; Higentec Co. Ltd., Changsha, Hunan, 410125, China; Engineering Research Center of Education, Ministry for Germplasm Innovation and Breeding New Varieties of Horticultural Crops, Key Laboratory for Vegetable Biology of Hunan Province, College of Horticulture, Hunan Agricultural University, Changsha 410128, China; Engineering Research Center of Education, Ministry for Germplasm Innovation and Breeding New Varieties of Horticultural Crops, Key Laboratory for Vegetable Biology of Hunan Province, College of Horticulture, Hunan Agricultural University, Changsha 410128, China; Engineering Research Center of Education, Ministry for Germplasm Innovation and Breeding New Varieties of Horticultural Crops, Key Laboratory for Vegetable Biology of Hunan Province, College of Horticulture, Hunan Agricultural University, Changsha 410128, China; Institute of Vegetable Research, Hunan Academy of Agricultural Science, Changsha 410125, China; Higentec Co. Ltd., Changsha, Hunan, 410125, China; Engineering Research Center of Education, Ministry for Germplasm Innovation and Breeding New Varieties of Horticultural Crops, Key Laboratory for Vegetable Biology of Hunan Province, College of Horticulture, Hunan Agricultural University, Changsha 410128, China; Engineering Research Center of Education, Ministry for Germplasm Innovation and Breeding New Varieties of Horticultural Crops, Key Laboratory for Vegetable Biology of Hunan Province, College of Horticulture, Hunan Agricultural University, Changsha 410128, China; Higentec Co. Ltd., Changsha, Hunan, 410125, China; Institute of Vegetable Research, Hunan Academy of Agricultural Science, Changsha 410125, China; Engineering Research Center of Education, Ministry for Germplasm Innovation and Breeding New Varieties of Horticultural Crops, Key Laboratory for Vegetable Biology of Hunan Province, College of Horticulture, Hunan Agricultural University, Changsha 410128, China; Engineering Research Center of Education, Ministry for Germplasm Innovation and Breeding New Varieties of Horticultural Crops, Key Laboratory for Vegetable Biology of Hunan Province, College of Horticulture, Hunan Agricultural University, Changsha 410128, China; Engineering Research Center of Education, Ministry for Germplasm Innovation and Breeding New Varieties of Horticultural Crops, Key Laboratory for Vegetable Biology of Hunan Province, College of Horticulture, Hunan Agricultural University, Changsha 410128, China; Engineering Research Center of Education, Ministry for Germplasm Innovation and Breeding New Varieties of Horticultural Crops, Key Laboratory for Vegetable Biology of Hunan Province, College of Horticulture, Hunan Agricultural University, Changsha 410128, China; Engineering Research Center of Education, Ministry for Germplasm Innovation and Breeding New Varieties of Horticultural Crops, Key Laboratory for Vegetable Biology of Hunan Province, College of Horticulture, Hunan Agricultural University, Changsha 410128, China; Engineering Research Center of Education, Ministry for Germplasm Innovation and Breeding New Varieties of Horticultural Crops, Key Laboratory for Vegetable Biology of Hunan Province, College of Horticulture, Hunan Agricultural University, Changsha 410128, China; Engineering Research Center of Education, Ministry for Germplasm Innovation and Breeding New Varieties of Horticultural Crops, Key Laboratory for Vegetable Biology of Hunan Province, College of Horticulture, Hunan Agricultural University, Changsha 410128, China; Engineering Research Center of Education, Ministry for Germplasm Innovation and Breeding New Varieties of Horticultural Crops, Key Laboratory for Vegetable Biology of Hunan Province, College of Horticulture, Hunan Agricultural University, Changsha 410128, China

## Abstract

Cytoplasmic male sterility (CMS) is pivotal in plant breeding and widely employed in various crop hybrids, including pepper. However, the functional validation of the restorer of fertility (*Rf*) gene in pepper has been lacking until now. This study identifies and characterizes *CaRf*, a single dominant locus crucial for restoring CMS in the pepper strong recovery inbred line Zhangshugang. The *CaRf* gene encodes a mitochondria-targeted pentatricopeptide repeat protein, validated through the induction of male sterility upon its silencing in hybrid F_1_ plants. To enhance pepper breeding efficiency, 176 important pepper breeding parent materials were resequenced, and a PepperSNP50K liquid-phase breeding chip was developed, comprising 51 172 markers. Integration of *CaRf* functional characterization and PepperSNP50K facilitated the development of a high-quality red pepper hybrid. These findings provide significant insights and practical strategies for advancing molecular-designed breeding in peppers.

## Introduction

Cytoplasmic male sterility (CMS) is a maternally inherited phenomenon prevalent in higher plants that hinders the production of functional pollen grains [1], thereby promoting genetic diversity through facilitated cross-pollination. Specific nuclear fertility restorer genes (*Rf*) [2, 3] counteract mitochondrial sterility genes, reinstating fertility and facilitating efficient hybrid seed production [4]. Compared with nuclear sterility systems, CMS/*Rf* breeding ensures 100% male-sterile offspring, significantly reducing labor costs in breeding programs [5, 6]. Various sterile lines have been identified in rice, including CMS-FA/*OsRf19* [7], CMS-BT [8], CMS-HL [9], CMS-LD [10], CMS-CW [11, 12], CMS-WA [13], CMS-RT102 [14], CMS-RT98 [15], CMS-D1 [16], and CMS-TA [17], all associated with chimeric open reading frames (ORFs) originating from mitochondrial genome rearrangements. For example, the CMS-WA line’s *WA352* gene comprises three mitochondrial fragments, *orf284*,* orf224*, and *orf288*, and a short sequence of unknown origin [13]. The discovery and utilization of rice CMS and *Rf* genes have greatly advanced international studies on hybrid rice and laid a crucial theoretical and practical foundation for mining CMS and *Rf* genes in other crops, including maize [18–21], wheat [22], barley [23], oilseed rapeseed [24], the fiber crop cotton [25], sugar beet [26], Chinese cabbage [27], radish [28], and onion [29].

*Capsicum*, an important member of the Solanaceae family, five major species of cultivated peppers: *C. annuum*, *C. frutescens*, *C. chinense*, *C. baccatum*, and *C. pubescens*. Among them, *C. annuum* is globally the most widely cultivated and abundant variety. In China, hybrid pepper varieties represent over 80% of the total market production [30]. Since the release of the pepper whole-genome sequence [31, 32], several genetic linkage maps have been established via whole-genome resequencing, facilitating the development of highly efficient molecular markers to identify *Rf* genes across different pepper populations. To date, numerous *Rf* genes in pepper have been mapped to contiguous regions on chromosome 6, with several candidate genes identified [5, 33–37]. Most of these *Rf* genes encode pentatricopeptide repeat (PPR) proteins, one of the largest protein families in embryophytes. PPR proteins are tandems of degenerate 35-amino-acid motifs, which are eukaryotic-specific RNA-binding elements affecting gene expression in organelles, particularly mitochondria. PPR proteins’ biological and molecular functions have been extensively explored and are highly correlated with fertility recovery [38]. In general, PPR proteins restore plant fertility by suppressing the production of mitochondrial CMS-inducing proteins through reduction of mitochondrial CMS-related gene transcription or by binding to CMS-related transcripts to inhibit mRNA translation [2, 38]. The PPR gene, as a candidate *Rf* gene in pepper, exhibits genotype-specific characteristics due to varying genetic backgrounds.

To enhance efficient pepper breeding, modern molecular breeding technologies need to be integrated into production. Over the past 5 years, numerous high-quality genome and resequencing projects have provided comprehensive insights into the structure and genetic diversity of the chili pepper genome. Projects such as Ca_59 [39], Zhangshugang [40], and CaT2T [41] have assembled valuable references for mining essential agronomic traits in pepper. These initiatives have identified millions of polymorphisms, including millions of single-nucleotide polymorphisms (SNPs), across the pepper genome, laying a robust foundation for developing a high-throughput genotyping system critical for gene identification and molecular breeding. Various SNP detection platforms have gained popularity as indispensable tools for genotyping. Liquid-phase microarray technology utilizes synthesized probes specific for multiple target sequences located in diverse genomic regions for liquid-phase hybridization capture enrichment. This approach has been successfully applied in breeding microarrays for various crops. For instance, Illumina Infinium BeadChip technology has led to the development of RiceSNP50 and RICE6K for rice [42, 43] and MaizeSNP50 for maize [44]. Recently, a target capture sequencing SNP genotyping platform has been developed for genetic analysis and genomic breeding in canola, demonstrating its effectiveness on double haploid (DH) populations [45]. The study highlighted that using mSNPs and their haplotypes instead of single SNP markers in solid-phase microarrays improves detection efficiency [45]. Moreover, liquid-phase microarrays are noted for their cost-effectiveness and user-friendly nature. In our study, we developed a new high-density SNP array, PepperSNP50K, for genotyping chili peppers by sequencing 176 samples of commonly used breeding materials in chili peppers with 10× coverage, yielding 51 172 SNPs. To further facilitate pepper breeding using the CMS/*Rf* breeding system, we identified the novel restorer gene *CaRf* in Zhangshugang and verified its functionality. The development of PepperSNP50K enhances the feasibility of molecular design breeding in peppers, promising significant advancements in pepper genetic improvement and breeding efficiency.

## Results

### Phenotypic and genetic analysis of CMS line 9704A and restorer line Zhangshugang

**Figure 1:**
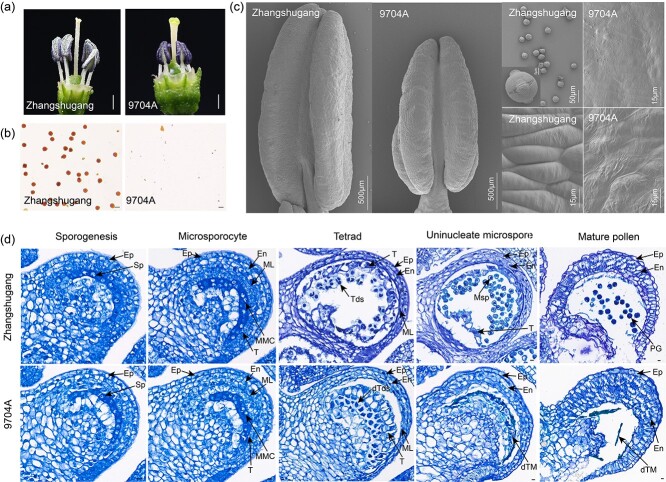
Phenotypes of Zhangshugang and 9704A. **a** Mature anther phenotypes of Zhangshugang and 9704A. Scale bars, 1 mm. **b** Pollen grains of Zhangshugang and 9704A stained with I_2_-KI. Scale bars, 50 μm; **c** Scanning electron microscopic analysis of anthers and pollen grains from Zhangshugang and 9704A at the mature pollen stage. **d** Sectional observation of anthers from Zhangshugang and 9704A at five different development stages. Scale bars, 10 μm. Sp, sporogenous cells; Ep, epidermis; En, endothecium; ML, middle layer; T, tapetum; MMC, microspore mother cells; Tds, tetrads; dTds, death tetrads; Msp, microspores; dTM, dead tapetum and microspores; PG, pollen grains.

We conducted phenotypic and genetic analyses of the male-sterile CMS line 9704A and the male-fertile restorer line Zhangshugang. Zhangshugang exhibited the ability to self-pollinate and produce healthy pepper fruits, whereas 9704A was observed to be completely sterile, characterized by shrunken small anthers devoid of visible pollen grains (Fig. 1a). In contrast, Zhangshugang displayed fully fertile characteristics with abundant pollen grains on its dehiscent anthers during flowering. Microscopic examination further revealed that pollen grains from Zhangshugang were well-developed and exhibited normal germination ability, whereas 9704A exhibited no pollen grains and only residual anther tissues (Fig. 1b). Comparative analysis indicated that the anthers of Zhangshugang were significantly larger than those of 9704A. Additionally, the anther wall of 9704A appeared smoother, with sparse arrangement of epidermal cells, and exhibited varying degrees of atrophy (Fig. 1c). Observation of flower buds at five different stages (Fig. 1d) revealed that anther abortion in 9704A commenced at the tetrad stage, characterized by abnormal expansion and premature degeneration of tapetum cells, resulting in insufficient nutrient supply during later microspore development, eventual microspore vacuolation, and death [46]. All F_1_ individuals resulting from the cross between 9704A and Zhangshugang were male-fertile. A fertility survey and statistical analysis of 1290 F_2_ individuals showed a separation ratio of male-fertile to sterile plants of 3:1 (χ^2^ = 1.266) (Supplementary Data Table S1), confirming that a single gene regulates fertility restoration in 9704A.

### Fine mapping of the *CaRf* locus

**Figure 2:**
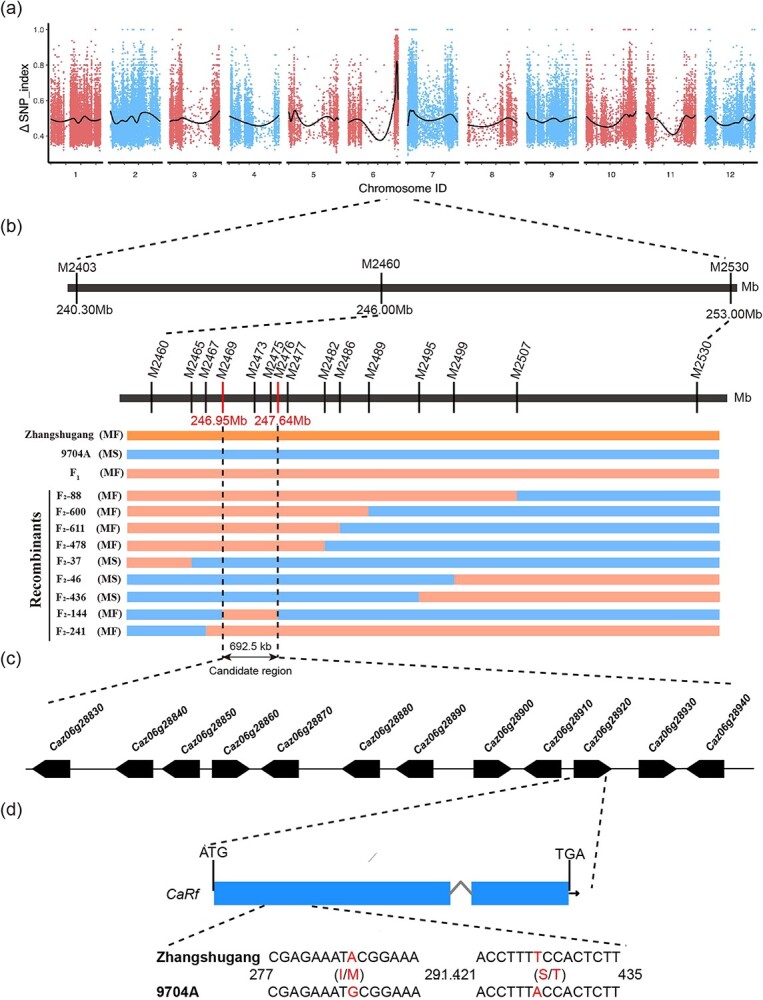
Mapping of the *CaRf* gene. **a** Distribution of SNP index on chromosomes. **b** Fine mapping of *CaRf* locus. MS, male-sterile; MF, male-fertile; **c** Genes within the candidate interval. Genes on the negative and positive strands are indicated on the left and right, respectively. **d** Predicted structure of the CaRf-coding protein sequence and alignment of the CaRf-coding protein sequence in Zhangshugang and 9704A.

We constructed bulked segregant analysis (BSA-seq) by sequencing bulk1 male-fertile (81.97 Gb at 27.11× coverage) and bulk2 male-sterile (78.32 Gb at 25.90× coverage), achieving >99% coverage of the whole genome (Supplementary Data Table S2). A total of 634 273 SNP loci were screened based on the filtering criteria. Using a sliding window approach with a window size of five SNPs and a step size of one SNP, we calculated the maximum allele frequency and ΔSNP index value across the genome (Fig. 2a). The analysis revealed a prominent peak region with a ΔSNP index value of 0.5–1 spanning the 240.00–253.00 Mb region on chromosome 6, indicating that this region harbors the candidate *CaRf* locus (Fig. 2b).

For fine mapping of *CaRf*, we designed 16 pairs of penta-primer amplification refractory mutation system (PARMS) markers within the candidate interval, with more markers covering the peak region (Supplementary Data Table S3). These markers were genotyped in 1290 F_2_ individuals from the 9704A × Zhangshugang cross, identifying 72 recombinant F_2_ plants between PARMS markers M2465 and M2482 (Supplementary Data Table S4). Notably, M2473 and M2475 co-segregated with fertility traits, confirming their complete linkage to the *CaRf* locus. Selected PARMS markers (M2469, M2473, M2475, M2476, M2477, and M2482) were further validated by genotyping in five restorer lines, three maintainer lines, and five sterile lines. The genotypes of sterile and maintainer lines were consistently *rfrf*, where restorer lines showed the *RfRf* genotype. PARMS markers M2473 and M2475 exhibited a coincidence rate of 100% (Supplementary Data Table S5). Subsequently, genotyping results from the 16 pairs of PARMS markers were used to construct a detailed genetic linkage map. Annotation of this interval against the reference genome identified 12 genes (Supplementary Data Table S6) spanning a physical distance of 692.5 kb (Fig. 2c). Notably, among these genes, *Caz06g28910*, *Caz06g28920*, and *Caz06g28930* encode PPR proteins. Sequencing these three PPR genes across different three-line materials revealed missense mutations in the *Caz06g28920* gene’s first CDS region [Chr06:247585338–247586517 (+ strand)] at positions 285 (A/G, isoleucine/methionine) and 427 (T/A, serine/threonine) (Fig. 2d, Supplementary Data Fig. S1). Further analysis using I-TASSER (https://zhanggroup.org/I-TASSER) [47] predicted structural differences in the Caz06g28920 protein between the restorer and sterile lines (Supplementary Data Fig. S2). These predictions highlighted three major differences between the two mutant proteins, suggesting that the observed missense mutations may influence protein structure and function. Future investigations using stable transgenic and protein experiments in peppers are warranted to elucidate the precise molecular mechanisms underlying these variations.

**Figure 3:**
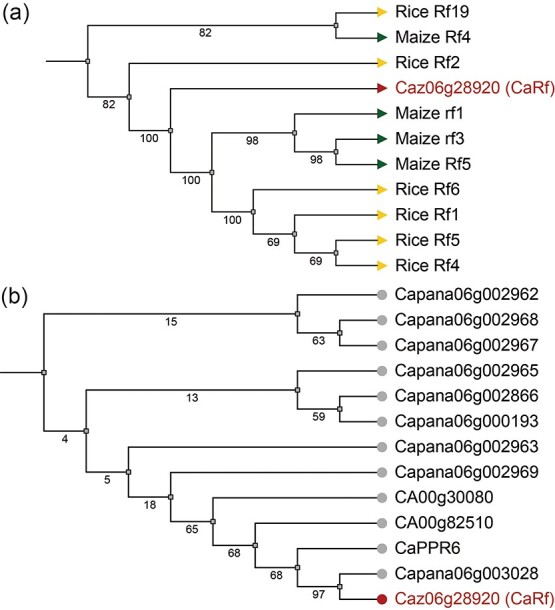
Candidate *Rf* genes reported in pepper and cloned *Rf* genes in rice and maize. **a** Sequence similarity between *CaRf* and cloned *Rf* genes in rice and maize. **b** Sequence similarity between *CaRf* and previous candidate *Rf* genes in pepper.

Furthermore, phylogenetic analysis of the protein sequences of identified restorer genes in various pepper genomes and the candidate gene *CaRf* revealed that *Caz06g28920* represents a novel restorer gene, with the highest homology to *Capana06g003028* [34] and *CaPPR6* [5], which are located in the distal region of pepper chromosome 6 and encode PPR proteins (Fig. 3a). Additionally, we compared the *Rf* genes with six cloned *Rf* genes from rice, which are suitable for different CMS types, and four cloned *Rf* genes from maize, highlighting their classification within the PPR protein family. This analysis demonstrated that the protein sequences of *CaRf* are closely related to those in maize (Fig. 3b).

### Gene expression analysis and subcellular localization a strong candidate male fertility restoration gene

**Figure 4:**
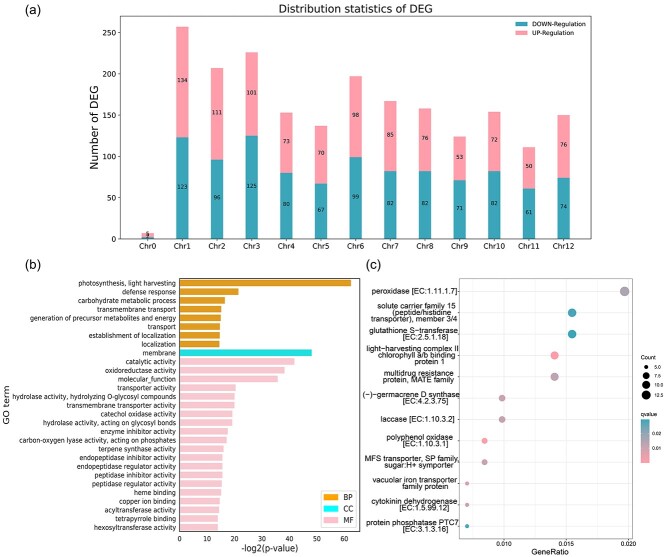
Comparative transcriptome analysis of male-fertile (MF; Zhangshugang) and male-sterile (MS; 9704A) anthers at the tetrad stage. **a** Number of DEGs identified based on the transcriptome analysis. ‘UP-regulation’ refers to DEGs with higher FPKM (fragments per kilobase of exon per million fragments mapped) values in MF anthers compared with MS anthers, while the ‘DOWN-regulation’ pertains to DEGs with lower FPKM values in MF anthers relative to MS anthers. DEGs were identified with a Q-value <0.01 and | log_2_(MF/MS) | > 1. **b** Classification of DEGs between MS and MF anthers based on GO terms, revealing three main categories: biological process (BP), cellular component (CC), and molecular function (MF). **c** Classification of DEGs between MS and MF anthers based on KEGG pathways, demonstrating their involvement in 12 pathways.

**Figure 5:**
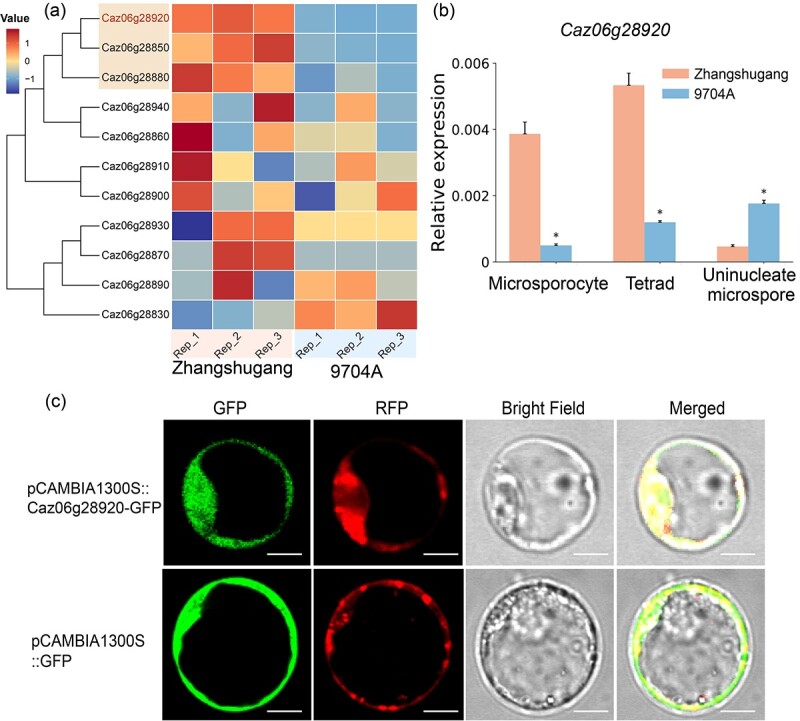
Gene expression and subcellular localization analysis. **a** Expression heat map of genes in the candidate interval at the tetrad stage of bud development in 9704A and Zhangshugang. **b** Relative *Caz06g28920* expression levels at different stages of flower bud development: microsporocyte, tetrad, and uninucleate microspore stages, analyzed using quantitative RT–PCR. **c** Subcellular localization of Caz06g28920 in rice protoplasts: co-localization of Caz06g28920-GFP and Mito-Tracker Red CMXRos-RFP in the mitochondrion. *P < 0.05, **P < 0.01. GFP, green fluorescent protein; RFP, Mito-Tracker Red CMXRos-RFP (red). Scale bars, 10 μm.

To further identify candidate *CaRf*, we conducted transcriptome analysis of flower buds at the tetrad stage from Zhangshugang and 9704A using RNA sequencing technology. Differential expression analysis of the entire pepper gene repertoire between Zhangshugang and 9704A revealed 2048 differentially expressed genes (DEGs). Among them, 1004 were up-regulated, and 1044 were down-regulated in Zhangshugang compared with 9704A (Fig. 4a). We examined the distribution of these DEGs across various chromosomes, noting Chr1 had the highest number and Chr11 the lowest. Gene Ontology (GO) functional annotation and enrichment analyses indicated that the majority of these DEGs were enriched in terms associated with CMS, including hormone level regulation, oxidoreductase activity, and cell wall macromolecular metabolism. CMS can arise from disruptions in nutrient synthesis and metabolism, energy metabolism, reactive oxygen species metabolism, and hormone synthesis and metabolism. GO enrichment analysis provided insight into the specific biological processes influencing pollen fertility and highlighted key DEGs responsible for 9704A’s sterile phenotype (Fig. 4b). Kyoto Encyclopedia of Genes and Genomes (KEGG) pathway analysis of DEGs revealed that these DEGs are mainly involved in pathways related to protein synthesis, such as ‘light-harvesting complex’ and ‘vacuolar iron transporter family protein’, as well as enzymes crucial for plant fertility, including ‘peroxidase’, ‘glutathione *S*-transferase, ‘polyphenol oxidase’, and ‘laccase’ (Fig. 4c). These protein complexes and enzymes likely play essential roles in pathways affecting pollen development and fertility. Further exploration within the candidate interval identified three DEGs (Table 1), including the PPR gene *Caz06g28920*. Its expression levels in flower buds at the tetrad stage from Zhangshugang were significantly higher compared with 9704A (Fig. 5a). Validation using qRT–PCR confirmed elevated expression of *Caz06g28920* in microsporocyte, tetrad, and uninucleate stages in Zhangshugang relative to 9704A (Fig. 5b).

**Table 1:** Statistics of DEGs in the candidate interval.

Gene ID	Annotation	log_2_*F* (MF/MS)	*P*-value
*Caz06g28850*	Protein NTM1-like 9	3.01	1.54E−04
*Caz06g28880*	Putative late blight resistance protein homolog R1A-3	2.04	2.24E−06
*Caz06g28920*	Pentatricopeptide repeat protein	4.01	5.26E−11

According to Zhangshugang genome’s annotation, three PPR protein genes were identified within the candidate interval. Initially, we extracted the protein sequences of these three genes and predicted their subcellular localization using Euk-mPLoc 2.0 (http://www.csbio.sjtu.edu.cn/bioinf/euk-multi-2/). The prediction indicated that Caz06g28910, Caz06g28920, and Caz06g28930 are located in the cytoplasm, mitochondria, and nucleus, respectively. During the amplification of these three PPR genes, we found potential annotation error in *Caz06g28910*. The Zhangshugang genome annotation placed this gene on the negative strand of chromosome 6 (Chr06:247554451–247574842), spanning a complex structure with a length of 20 392 bp (Supplementary Data Fig. S3). Analysis based on the typical characteristics of the PPR protein family, which lacks introns and exhibits highly conserved domains [48], raised doubts about the validity of *Caz06g28910* as a genuine gene. Global alignment with the gapless genome CaT2T [41] revealed that *Caz06g28910* aligned with seven genes in the CaT2T genome (Supplementary Data Fig. S4), none of which matched a PPR protein gene of similar length in the CaT2T genome annotation. This discrepancy suggests that *Caz06g28910* might be an annotation error in the Zhangshugang genome, rectified in the current CaT2T genome annotation.

To verify the subcellular localization of the remaining two PPR genes within the interval, we constructed pCAMBIA1300 vectors expressing Caz06g28920-GFP and Caz06g28930-GFP fusion proteins under the control of the 35S promoter. These constructs were introduced into rice protoplasts along with mitochondria-specific marker Mito-Tracker Red CMXRos-mCherry (RFP) or with nucleus-specific marker mCherry-fused Ghd7. As shown in Fig. 5c, the fluorescent microscopy results confirmed that Caz06g28920-GFP was co-localized well with the mitochondrial marker Mito-Tracker Red CMXRos-mCherry (RFP), indicating its mitochondrial localization. In addition, Caz06g28920-GFP also showed partial localization in the cytoplasm, suggesting that it is synthesized in the cytoplasm and functions in the mitochondrion. Similarly, Caz06g28930-GFP co-localized effectively with the nuclear marker Ghd7-mCherry (RFP), confirming its nuclear localization (Supplementary Data Fig. S5). Therefore, the gene expression patterns and subcellular localization results strongly support *Caz06g28920* as a candidate *Rf* gene in pepper.

### Down-regulation of *CaRf* significantly decreases pollen fertility

**Figure 6:**
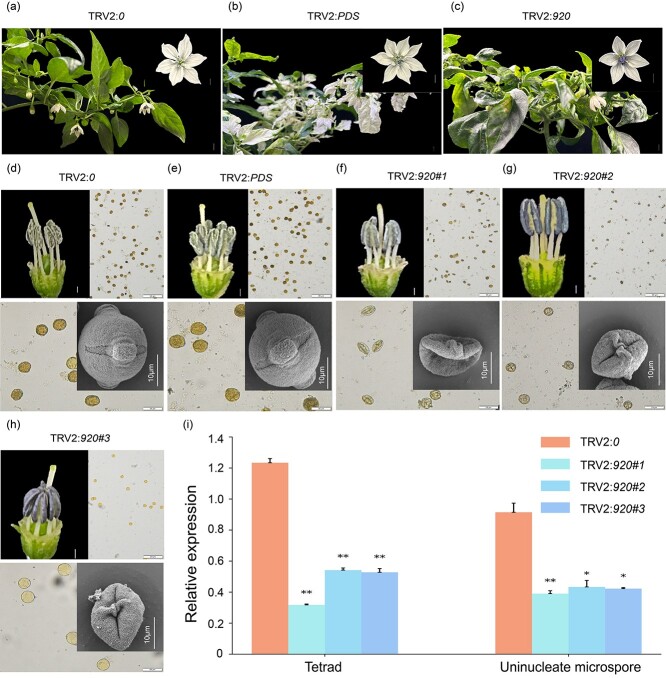
Functional verification of *Caz06g28920* through VIGS in F_1_ (9704A × Zhangshugang) plants. **a**–**c** Phenotypic comparison of TRV2:*0* (empty vector control), TRV2:*PDS* (phytoene desaturase), and TRV2:*920* plants. Scale bars: 1.0 cm (**a**, **b**) and 0.5 cm (**c**). **d**, **e** Anther dehiscence and pollen grains of TRV2:*0* and TRV2:*PDS* plants in F_1_ generation. Scale bars: 1 mm. **f**–**h** Anther dehiscence and pollen grains of *Caz06g28920* down-regulated TRV2:*920#1*, TRV2:*920#2*, and TRV2:*920#3* plants in F_1_ generation. Scale bars for dehisced anthers and pollen grains: 1 mm. **i**  *Caz06g28920* expression levels in virus-induced F_1_ plants; gene expression was analyzed using qRT–PCR on flower buds at tetrad and uninucleate microspore stages. Relative expression levels in TRV2:*920#1*, TRV2:*920#2*, and TRV2:*920#3* plants were compared with TRV2:*0*. *P < 0.05, **P < 0.01 (*t*-test). Relative expression levels are presented as mean  ± standard deviation, *n* = 3.

To substantiate the involvement of *Caz06g28920* in male fertility regulation in pepper, we employed the tobacco rattle virus 2 (TRV2)-based virus-induced gene silencing (VIGS) method to down-regulate its expression. As shown in Fig. 6i, *Caz06g28920* expression was significantly down-regulated in *Caz06g28920*-silenced plants compared with TRV2:*0* (empty vector) plants. A comparison with TRV2:*0* plants revealed distinctive outcomes: TRV2:*PDS* (positive control) displayed noticeable photobleaching in their leaves ~20 days post-inoculation (Fig. 6a–c). Upon anther dehiscence, TRV2:*920*-infiltrated plants exhibited a striking reduction in pollen viability and a high incidence of malformed pollen grains, contrasting with the abundant and healthy pollen grains observed on TRV2:*0* plants. Microscopic inspection confirmed that TRV2:*0* anthers produced copious viable pollen grains, whereas TRV2:*920* anthers predominantly contained deformed and non-functional pollen grains (Fig. 6d–h). Pollen viability analysis further revealed a significant reduction in pollen viability in *Caz06g28920*-silenced plants compared with TRV2:*0* controls. Electron microscopy examination indicated elevated pollen development and diminished pollen grain size in *Caz06g28920*-silenced plants, confirming their male-sterile phenotype and demonstrating that *Caz06g28920* down-regulation could induce male sterility in pepper. Importantly, qRT–PCR experiments verified that expression levels of *Caz06g28910* (Supplementary Data Fig. S6a and b) and *Capana06g003028* (Supplementary Data Fig. S6c and d) remained unaffected in *Caz06g28920*-silenced plants.

### Design and characteristics of PepperSNP50K

Positioning and functionally validating the *CaRf* gene is crucial for efficient pepper breeding in the CMS/*Rf* system. To streamline production, precise physical localization of *CaRf* and the development of a comprehensive genotyping tool are essential. While molecular markers have been developed, their widespread application is hampered by inefficiencies and limited background selection capabilities. Integrating advanced molecular breeding technologies is therefore imperative to enhance pepper breeding efficiency.

We designed a pepper whole-genome SNP array designed for efficient progeny screening, focusing on genetic background selection and targeted gene genotyping. Initially, we gathered 176 pepper parent materials with diverse agronomic traits pivotal for China’s pepper breeding. Subsequent whole-genome resequencing of these materials provided crucial data for SNP identification. High-density SNP arrays were then crafted to facilitate high-throughput genotyping. Each candidate SNP underwent rigorous evaluation of the position, average polymorphic information content (PIC), and minor allele frequencies (MAFs) to ensure the array’s quality and applicability across various research and breeding programs. Our SNP selection process involved a meticulous algorithm that culled 47 589 SNP loci, 3194 multiple dispersed nucleotide polymorphism (MNP)-tagged loci, and 389MNP-tagged loci association with key pepper agronomic traits from over a million candidates. These loci were chosen for their representativeness, polymorphism, and uniform distribution across chromosomes. Additionally, Kompetitive Allele-Specific PCR (KASP) markers developed in this study were incorporated to analyze *CaRf* genotypes.

**Figure 7:**
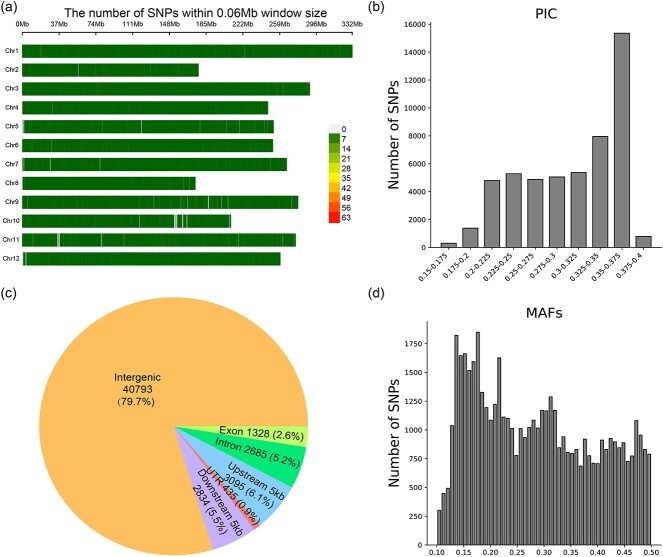
Design and characteristics of PepperSNP50K. **a** Distribution of SNPs across the entire pepper genome. **b** PICs of the selected SNPs. **c** Localization of selected SNPs within gene regions and across chromosomes. **d** MAFs of the selected SNPs.

Utilizing Genotyping by Pinpoint Sequencing (cGPS) technology, we successfully synthesized the PepperSNP50K liquid microarray, encompassing 51 172 SNPs with robust polymorphism and even distribution across the chromosome (Fig. 7a). Statistical details of SNP positions are cataloged in Supplementary Data Table S7. Mapping these SNPs to the pepper Zhangshugang genome assembly revealed an average inter-SNP distance of 60 kb. Analysis of SNPs within PepperSNP50K yielded an average PIC of 0.30, crucial for discerning genetic variations (Supplementary Data Table S2). The distribution of PIC values is illustrated in Fig. 7b, underscoring the array’s utility in diverse breeding contexts.

Within PepperSNP50K, 21.3% of SNPs were located within the gene region, encompassing the upstream and downstream 2 kb of the gene, UTRs, exons, and introns (Fig. 7c). These SNPs hold significant potential to influence gene function and are usually more informative. While this percentage is lower compared with 68% in RiceSNP50 [42], SNPs in PepperSNP50K are evenly distributed across chromosomes. This distribution ratio is optimal considering the larger size of the pepper reference genome, which is 7–8 times that of the rice reference genome, and the relatively smaller proportion of gene regions in peppers. To ensure broad applicability within the pepper community, our initial SNP selection prioritized those with high MAFs within each linkage disequilibrium (LD) cluster. As depicted in Fig. 7d, all SNPs on PepperSNP50K exhibited MAFs > 0.1, with a mean MAF of 0.285 (Supplementary Data Table S8). This indicates a high likelihood of identifying enough informative SNPs across any two chili pepper plants.

PepperSNP50K probes are adept for use in liquid-phase breeding microarrays, enabling rapid and accurate detection of numerous genes through extreme mixed-pool sequencing. Localization results obtained using the PepperSNP50K liquid-phase microarray align with those from traditional BSA-seq resequencing, pinpointing candidate intervals predominantly at the end of chromosome 6 (Supplementary Data Fig. S7), indicating the effectiveness of PepperSNP50K in precise pepper genotyping.

### Application of PepperSNP50K and CMS/*Rf* system in hybrid pepper breeding

**Figure 8:**
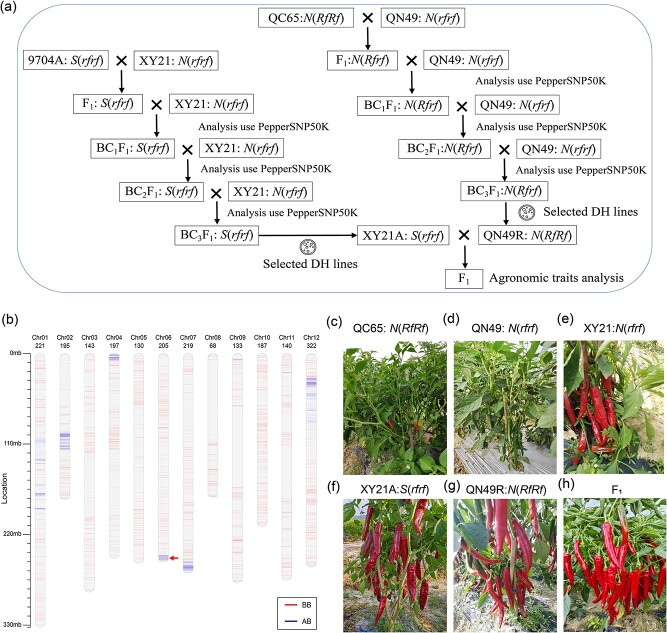
Breeding application of CMS/*CaRf* system. **a** Development of CMS hybrids for breeding programs. XY21(*N*(*rfrf*)) and QN49(*N*(*rfrf*)) are both maintainer lines. The creation of a sterile line and a restorer line involved using the sterile line 9704A and the restorer line QC65, respectively. Through hybridization and multiple recurrent hybridizations, PepperSNP50K was used to analyze the background recovery rate of each generation and select the target genotype. Ultimately, the sister sterile line XY21A of XY21 and the new restorer line QN49R carrying homozygous disease resistance genes were bred. The breeding process included three backcrosses, self-breeding in one generation, and the creation of DH lines. The male parent (QC65) contains *CaRf* loci. **b** Genetic background analysis of QN49-*CaRf* restorer lines using PepperSNP50K array. Short blue and red lines on the chromosome represent SNP sites of different genotypes. The red arrow indicates the position of *CaRf*. **c** Phenotype of QC65(*N*(*RfRf*)) in the field. **d** Phenotypes of QN49 in the field. **e** Phenotype of XY21-(*N*(*rfrf*)) in the field. **f** After multiple rounds of crosses with XY21, DH treatment, inoculation testing, and marker-assisted selection (MAS) in DH lines, the new male-sterile line XY21A was developed. **g** After multiple rounds of crosses with QN49, DH treatment, inoculation testing, and MAS in DH lines, the new restorer line QN49R, which introduced the pepper restorer gene, was selected. **h** Phenotype of commercial variety F_1_ bred through crossbreeding.

In pepper breeding, crossing the varieties XY21 and QN49 yields offspring with excellent traits. Using the KASP molecular markers developed in this study alongside PepperSNP50K, we identified genotypes of XY21(*N*(*rfrf*)) (Fig. 8e) and QN49(*N*(*rfrf*)); both are natural maintainer lines. In practical breeding applications, XY21 and QN49 are chosen as the female and male parents, respectively, due to their superior traits. To improve the efficiency of crossing between XY21 and QN49, we employed the three-line system involving 9704A/*CaRf* to create a new sterile line, XY21A(*S*(*rfrf*)). During the development of the male QN49R restorer line, we utilized the homozygous restorer line QC65(*N*(*RfRf*)) (Fig. 8c). Despite being a high-quality red pepper variety, the recurrent parent QN49 lacks the *CaRf* locus (Fig. 8d). Utilizing PepperSNP50K, we screened individuals possessing restorer genes and achieving high background recovery rates. Subsequently, each backcross population was screened for phenotypes carrying the restorer gene *N*(*Rfrf*) (Fig. 8a). By the third generation of backcrossing, the background recovery rate between the backcross population and the original QN49, as detected by PepperSNP50K, ranged from 78.26% to 94.74%, meeting pepper breeding standards (Fig. 8b). We cultured anthers *in vitro* from the selected plants that met the expectations and treated them with colchicine to generate a DH line with homozygous genotype. Individual plants with genotype *N*(*RfRf*) were identified as a new restorer line, QN49R, exhibiting basic agronomic traits consistent with the original QN49 (Fig. 8g) (Table 2).

**Table 2:** Agronomic performance of various varieties.

Variety	Type	Germination rate (%)	First flower node (cm)	Plant height(cm)	Effective number of branches	Single fruit quality (g)	Longitudinal diameter of fruit (cm)	Fruit cross diameter (cm)	Fruit type index	Pulp thickness (cm)	Yield per plant (g)	Capsanthin content
XY21	Maintainer line	95 ± 0.8	12 ± 0.5	65 ± 3.3	10 ± 1.5	22 ± 1.1	13.5 ± 0.5	2.5 ± 0.1	5.4 ± 0.5	0.3 ± 0.04	500 ± 10	21 ± 1.3
XY21A	Male-sterile line	95 ± 0.6	12 ± 0.8	65 ± 4.2	10 ± 1.5							
QC65	Restorer line	95 ± 0.9	15 ± 0.6	62 ± 2.7	10 ± 1	5 ± 0.3	5.1 ± 0.2	1 ± 0.1	5.1 ± 0.5	0.15 ± 0.01	150 ± 6.3	10 ± 0.6
QN49	Maintainer line	95 ± 1.1	15 ± 0.7	85 ± 3.6	7 ± 1.5	35 ± 2.5	18 ± 1.5	2.5 ± 0.1	7.4 ± 0.5	0.3 ± 0.03	585 ± 12.6	24 ± 1.1
QN49R	Restorer line	95 ± 1.0	15 ± 0.5	85 ± 3.1	7 ± 1.5	36 ± 1.8	19 ± 1.2	2.5 ± 0.2	7.6 ± 0.6	0.3 ± 0.04	590 ± 15.1	25 ± 1.5
*F*_1_		95 ± 10.8	14 ± 0.6	77 ± 4.1	9 ± 1	33 ± 1.2	16 ± 0.8	3 ± 0.2	5.3 ± 0.5	0.3 ± 0.03	610 ± 12.3	17.51 ± 1.3

Values are presented as mean ± standard deviation, *n* = 3

The female parent XY21(*N*(*rfrf*)) functions as a natural maintainer line, necessitating the creation of a corresponding sterile line, XY21A(*S*(*rfrf*)). To achieve this, we employed the sterile line 9704A, which is currently the most widely used in pepper genetic breeding, as the female parent for hybridization. All offspring did not separate and exhibited the *S*(*rfrf*) genotype. Subsequently, these offspring were used as female parents in recurrent hybridization with XY21 as the male parent. After three cycles of hybridization, the background recovery rate reached the standard. A homozygous line was then developed through DH, resulting in the creation of the sister line XY21A(*S*(*rfrf*)) derived from the original XY21 (Fig. 8f), exhibiting basic agronomic traits consistent with XY21 (Table 2). In this study, the commercial variety produced by crossing the newly created sterile line XY21A(*S*(*rfrf*)) and the restorer line exhibited excellent heterosis traits (Fig. 8h), demonstrating significant generalizability and economic value. Supplementary Data Table S10 lists detailed information regarding the timing and location of this molecular breeding experiment.

## Discussion

### Identification and functional verification of *CaRf* provide a theoretical basis for molecular design breeding of pepper

CMS is a phenotype resulting from interactions between cytoplasmic and nuclear genomes, influenced by multiple genes and various modification mechanisms. Before the complete sequence of the pepper genome was published, studies on restoring fertility focused primarily on mapping major quantitative trait loci (QTLs) associated with pepper fertility restoration, predominantly on chromosome 6 [5, 49–51]. Advancements in high-throughput and third-generation sequencing technologies have since facilitated the higher-quality assembly of genomes from species with complex genetic structures, such as pepper [31, 32]. These genome assemblies, including references like Ca_59 [39] and Zhangshugang [40] in pepper, have provided valuable resources for further studies. Subsequently, multiple candidate *Rf* genes encoding PPR proteins have been identified across diverse pepper germplasms, with sequence identity exceeding 85% (Fig. 3a).

To facilitate pepper breeding using the CMS/*Rf* system, we identified novel male fertility restoration *Rf* genes in *C. annuum* L. using the cytoplasmic male sterile line 9704A and its corresponding restorer line Zhangshugang. We utilized the latest Zhangshugang genome sequence, assembled from third-generation sequencing data, as a reference for sequence alignment and genetically analyzed the fertility of 9704A across multiple genetic populations. Our findings revealed that fertility restoration is regulated by a single dominant gene, designated *CaRf*. We performed BSA-seq analysis on segregating F_2_ individuals and employed PARMS markers to construct a genetic map for fine mapping *CaRf*. Our investigation pinpointed *CaRf* to the genomic region spanning 246 954 384–247 646 884 bp on chromosome 6, where it co-segregates with two PARMS markers. Subcellular localization analysis revealed that Caz06g28920-encoded PPR protein resides in the mitochondrion (Fig. 5c), aligning with previous findings that OsRf19-encoded PPR protein in rice also localizes in the mitochondrion [7].

**Figure 9:**
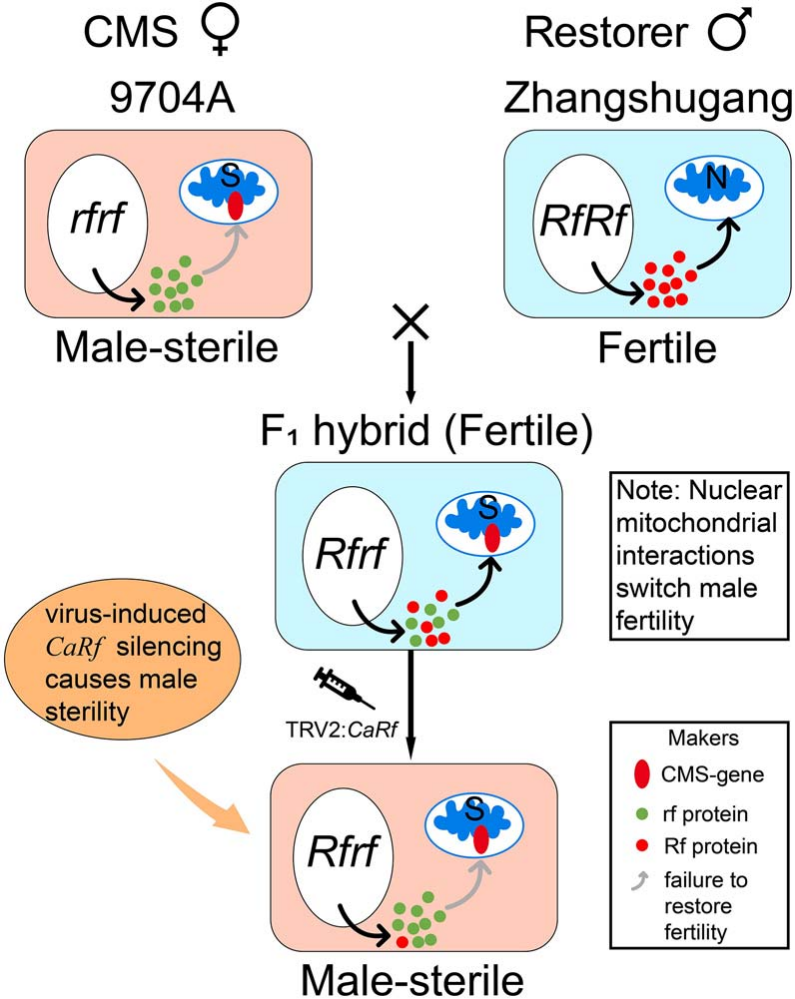
Proposed model illustrating the functional validation mechanism for fertility restorer genes in three-line hybrid seed production using F_1_ (9704A × Zhangshugang) hybrids.

In pepper, while more than 10 CMS restorer genes have been identified through genetic mapping in various restorer lines, experimental validation of these genes remains insufficient. The CMS line used for three-line hybrid seed production produces an F_1_ hybrid with a CMS gene in the mitochondria and a restorer gene in the nucleus. Based on our findings, we proposed a model illustrating that the mechanism of these F_1_ hybrids can efficiently verify the reliability of restorer genes (Fig. 9). In our VIGS experiment, we silenced the expression of the putative restorer gene in the nucleus in the F_1_ hybrid (9704A × Zhangshugang) (Fig. 9). Compared with control plants, the silenced plants exhibited a substantial reduction in pollen viability and a high proportion of malformed pollen grains. These findings strongly support the notion that *CaRf* indeed functions as a credible restorer gene (Fig. 6).

There are missense mutations in the *Caz06g28920* gene in the restorer line Zhangshugang and the sterile line 9704A, which may lead to decreased expression of the *Caz06g28920* gene in the sterile line 9704A or differences in protein function. However, the causes of Caz06g28920 protein dysfunction in 9704A remain unclear. We conducted a VIGS experiment targeting the *Caz06g28920* gene in the first-generation hybrid of sterile line 9704A and restorer line Zhangshugang. The results showed that plants with effective silencing of *Caz06g28920* showed obvious pollen growth deformities and reduced pollen vitality compared with controls, suggesting that silencing *Caz06g28920* interrupts fertility recovery in the first-generation hybrid. Nevertheless, the specific underlying mechanism is complex and requires further elucidation. Future efforts will focus on creating a stable transgenic pepper strain carrying the *Caz06g28920* gene and identifying the sterility gene in sterile line 9704A to facilitate deeper molecular analysis.

Molecular marker-assisted selection technology significantly accelerates breeding timelines and improves breeding efficiency. The PARMS markers developed in this study, closely linked to the *Rf* gene, offer substantial benefits for molecular-assisted breeding in pepper. Previous studies have shown that the established pepper *Rf*-linked markers CRF-SCAR and G16-SCAR exhibit accuracies of 70.0–89.1% [5]

and 82.7% [52], respectively. Moreover, KSAP markers S1597 and S1609 demonstrate higher accuracy and success rates compared with traditional marker-assisted selection methods in peppers [53]. In this study, we developed and validated four *CaRf*-linked PARMS markers: M2469, M2473, M2475, and M2476. Our results indicate that M2473 and M2475 achieved 100% accuracy in genotyping and verification within the other three-line matching systems, while M2469 and M2476 achieved 66.67% accuracy. This underscores the strong application potential of M2473 and M2475 in pepper production, particularly in three-line hybrid breeding. These markers can expedite the identification and directional breeding of pepper cytoplasmic male-sterile lines and shorten the breeding duration for restorer and maintainer lines. Consequently, they facilitate the selection of new high-quality varieties.

Further investigations are warranted to assess the broader applicability of these PARMS markers in commercial CMS systems for marker-assisted pepper restorer line breeding and maintainer line transformation and selection and determine their feasibility in production applications. Genetic mapping of CMS restoration genes in pepper, coupled with elucidation of the molecular mechanisms underlying fertility restoration, will accelerate the adoption of the three-line matching method for producing hybrid seeds. These advancements hold significant theoretical implications for molecular breeding in pepper. Assuming a single restorer gene can effectively restore male fertility in hybrids without compromising hybrid performance, inheritance of such a gene will greatly simplify the breeding process for maintainer and restorer lines. This streamlined approach, particularly when combined with genomic selection technologies, promises to accelerate the development of hybrid pepper varieties.

### Development of PepperSNP50K represents a significant advancement in pepper molecular breeding tools

PepperSNP50K, designed in this study leveraging abundant pepper resequencing data, offers several advantages. SNP selection was vigorous, drawing from comprehensive resequencing datasets and filtering based on stringent criteria detailed in the Materials and methods section. This ensures PepperSNP50K’s reliability and high quality across various genotyping targets, incorporating considerations such as flanking sequence specificity, MAF, LD, and PIC. The array’s robustness in conjunction with the cGPS system makes it a valuable asset for the chili pepper community. Although whole-genome sequencing (WGS) remains flexible, its cost prohibits widespread adoption for deep resequencing in chili peppers with a complex 3G genome. PepperSNP50K offers a cost-effective alternative, being approximately one-third the cost of WGS while delivering comparable genotyping information. Its high-density format is particularly advantageous for efficiently genotyping large sample sets within short timeframes. PepperSNP50K’s utility spans diverse applications in pepper research and breeding. It effectively clusters pepper varieties based on genotypic data, facilitating genetic diversity analyses and parental selection, and enhancing overall breeding efficiency. Thus, PepperSNP50K represents a pivotal tool for advancing molecular design breeding in chili peppers.

Traditional breeding relies heavily on phenotypic observations and breeder experience, which can be challenging for traits such as disease resistance, root characteristics, and quality attributes. To address these challenges, there is a growing demand for high-throughput genotyping platforms. Molecular design breeding is considered the most efficient option for improving breeding methods. Array-based genotyping technologies play a crucial role in identifying genes or QTLs, optimizing genotypes, and designing efficient breeding strategies in molecular breeding [54–56]. Geneticists and breeders are increasingly adopting genome-wide selection strategies to improve crop efficiency and reduce genotyping costs [45]. These strategies have broad applications across crop species. The study successfully localized the CMS fertility restoration gene in peppers using PepperSNP50K, a finding consistent with BSA-seq localization. This proves the chip’s high accuracy in gene localization, affirming its utilization in molecular breeding of peppers. PepperSNP50K has demonstrated effectiveness in validating pepper varieties and advancing molecular breeding practices.

Through the development of PepperSNP50K for matching the fertility restorer gene in the 9704A sterile line and verifying its function, we have established a system conducive to rapid pepper breeding. By creating new three-line resources and cultivating varieties with excellent traits, we have demonstrated significant practical applications in pepper breeding and production. Compared with traditional breeding methods, DH technology offers the advantage of rapidly generating inbred lines [57], forming the basis of our accelerated pepper breeding platform. Leveraging the PepperSNP50K chip, CMS/*CaRf* system, and DH technology enables us to swiftly develop restorer line materials with excellent characteristics. To further shorten the overall pepper breeding cycle, it is necessary to conduct experiments and optimize parameters specific to pepper cultivation. Following this, we plan to establish a pepper speed breeding facility modeled after the rice (SpeedFlower) speed breeding facility. This facility will explore optimal light combinations, temperature, humidity levels, fertilizer ratios, and other components tailored to pepper growth [58]. Theoretically, by controlling the entire cycle from germination to harvesting within 3 months, we aim to significantly improve breeding efficiency, with the potential to complete breeding in as little as 1.5 years. This study represents a breakthrough in molecular breeding achieved through the functional identification of *CaRf* and the integration of PepperSNP50K and DH technology. By swiftly obtaining homozygous lines, we effectively shorten breeding time. As functional genes/QTLs are identified in pepper, PepperSNP50K can tap into additional functional sites for prospective selections in subsequent breeding efforts, including loci governing crucial agronomic traits such as disease resistance and fruit size. This approach enables efficient molecular design breeding of peppers, facilitates targeted trait aggregation breeding, and serves as a valuable reference for other horticultural crops.

## Materials and methods

### Plant materials

The CMS line 9704A and restorer line Zhangshugang were crossed to develop an F_2_ segregating population. A total of 1290 F_2_ plants were cultivated in a film greenhouse at Hunan Agricultural University. From this population, 20 fertile plants and 20 sterile plants were selected to form the fertile pool and sterile pool, respectively, for bulked-segregant analysis combined with whole-genome resequencing (BSA-seq). Additionally, six restorer lines, four maintainer lines, and five sterile lines from the Pepper Research Group of the College of Horticulture, Hunan Agricultural University, were used to validate the markers developed in this study.

### Observation of anther tissue microstructure

The microstructure of anther tissues was observed under an optical microscope and a scanning electron microscope. For the former, buds from 9704A and Zhangshugang at five different developmental stages were fixed in 5 ml of FAA solution containing 10% formalin, 5% glacial acetic acid, and 50% ethanol (Servicebio, Wuhan, China), dehydrated in a series of ethanol solutions with concentrations increasing until 100%, embedded in paraffin, and prepared as cross-sections using a pathological slicer (Leica, RM2016). The sections were completely dewaxed, stained with toluidine blue solution (ServiceBio, G1032, Wuhan, China) for 2–5 min, and observed under a Nikon optical microscope (Nikon Eclipse E100). For the latter, fresh anthers at the uninucleate microspore and mature pollen stages were collected from 9704A and Zhangshugang lines and fixed in 1% OsO_4_ in 0.1 M phosphate buffer (PB, pH 7.4) for 1–2 h at room temperature. After rinsing three times in 0.1 M PB (pH 7.4) for 15 min to remove traces of fixative, they were prepared as tissue blocks. After that, the blocks were washed again with 0.1 M PB (pH 7.4) three times for 15 min each and dried using a critical point dryer. Specimens were mounted on metallic stubs using carbon stickers, sputter-coated with gold for 30 s, observed under a scanning electron microscope, and photographed.

### Male fertility phenotyping

Plant fertility was assessed based on visual observation of the size of anthers and pollen grains. The fertile phenotype was characterized by plump anthers bursting with abundant pollen grains upon cracking, while the sterile phenotype exhibited withered anthers lacking pollen grains after cracking. In instances where fertility determination was inconclusive by visual inspection, anthers from three mature flowers were subjected to I_2_-KI staining and observed under a light microscope to determine pollen viability, thereby determining fertility status. All plants were investigated three times every 5 days, with four to six flowers examined each time. The segregation ratio in the constructed genetic population was analyzed using the χ^2^ test in Microsoft Excel (2019).

### Bulked-segregant analysis combined with whole-genome resequencing

The genomes of the pooled fertile and sterile samples were sequenced on an Illumina Novaseq 6000 system using the PE150 mode. Raw reads underwent quality control and adapter trimming using the Trimmomatic tool to generate clean reads [59]. Clean reads were aligned to the Zhangshugang reference genome [40] (http://ted.bti.cornell.edu/) using BWA software (Burrows–Wheeler Aligner, v0.7.12, http://ted.bti.cornell.edu/cgi-bin/pepper/index). Supplementary Data Table S4 lists the sequencing information. SNPs were identified using GATK (v3.2–2) software with specific criteria: (i) sequencing depth ≥5 for each SNP; (ii) base alignment quality value ≥20; and (iii) variant detection quality value ≥30. The SNP index was calculated using parameters including: (i) filtering out bulk1 (male-fertile) > 0.5 and bulk2 (male-sterile) < 0.3; (ii) sliding window at five SNPs; (iii) sliding at one SNP; and (iv) screening threshold set at 95% confidence level.

### Kompetitive Allele-Specific PCR marker development and genetic mapping

For genetic linkage analysis and fine mapping of *CaRf*, we utilized PARMS markers for SNP genotyping in 1290 F_2_. PARMS is a KASP-like fluorescent PCR analysis technique based on allele-specific amplification. Genomic DNA was extracted using the CTAB method from tender green leaves of parents and F_1_ and F_2_ individuals [60] and used for genotyping purposes. The online primer design software SNP Primer (www.snpway.com) was used to design marker primers (Supplementary Data Table S3), including a FAM-labeled and a HEX-labeled SNP allele-specific forward Primer A and Primer B, and a common reverse Primer C. According to the SNP typing results and plant fertility assessments, recombinant plants were selected from the F_2_ populations for genetic linkage analysis to construct the physical *CaRf* genetic map, thereby identifying candidate *CaRf* genes from the targeted interval.

### Quantitative real-time PCR

Total RNA was extracted from flower buds of Zhangshugang and 9704A lines at the pollen mother, tetrad, and mononuclear microspore stages using the Trizol UP kit (TransGen Biotech, Beijing, China). After treatment with DNase I (Thermo Fisher, San Jose, USA), RNA samples were reverse-transcribed into cDNA using the Revert Aid First Strand cDNA Synthesis Kit and subjected to qRT–PCR in a 20-μl reaction system containing 10 μl of 2x ChamQ Universal SYBR qPCR Master Mix (Vazyme, Nanjing, China). Each reaction was set up with three biological replicates and three technical replicates. The relative expression of genes was calculated using the ΔCt method [61] with pepper *UBI-3* [62] as the internal control. All primers are listed in Supplementary Data Table S9.

### Cloning of important genes in male-fertile and sterile plants

For gene cloning, we designed gene-specific primers using the Primer-BLAST tool on the NCBI website (https://www.ncbi.nlm.nih.gov/tools/primer-blast) (Supplementary Data Table S9). PCR amplification was conducted using the Phanta Max Super-Fidelity DNA Polymerase kit (Vazyme, Nanjing, China). After gel-purification using the gel recovery kit (Vazyme, Nanjing, China), PCR products were cloned into the pCE2 TA/Blunt-Zero vector (Vazyme, Nanjing, China) and transformed into Trans1-T1 phage-resistant competent cells (TransGen Biotech, Beijing, China). Three or four positive colonies from each sample were randomly selected and confirmed by Sanger sequencing. The assembled sequences were compared using SnapGene software.

### Phylogenetic tree analysis of restorer genes in pepper

The proteomic data for *C. annuum* L. (Zunla, CM334) were retrieved from NCBI (https://www.ncbi.nlm.nih.gov/). Multiple sequence alignments of genes of interest were conducted using Multiple Protein Sequence Alignment (MUSCLE). Phylogenetic trees were constructed using TreeBest software with the contiguous algorithm [63] and illustrated using the online tool iTOL (Interactive Tree Of Life; https://itol.embl.de/) [64].

### Collinearity analysis of genes encoded by Zhangshugang genome and CaT2T

CDS sequences and annotation files of the whole-genome coding genes of Zhangshugang and CaT2T were downloaded, formatted appropriately, and analyzed for collinearity using the JCVI library [65].

### Subcellular localization analysis of the PPR genes

The coding regions of *Caz06g28920* and *Caz06g28930* without the stop codon were amplified and cloned into the pCAMBIA1300S-eGFP vector, which had undergone modifications at multiple cloning sites (Supplementary Data Table S9). Mito-Tracker Red CMXRos-mCherry [66] was used as the mitochondrial marker, and Ghd7-RFP-mCherry [67] was used as the nucleus marker. Rice protoplasts were isolated from 12-day-old seedlings and subjected to transient expression assay following a previously described protocol [68]. Green fluorescent protein (GFP) and the mCherry fluorescent protein were observed and photographed using a confocal microscope (FV 1200; Olympus, Tokyo, Japan).

### RNA-seq and differential gene expression analysis in 9704A and Zhangshugang lines

Total RNA was extracted from flower buds of Zhangshugang and 9704A lines at the tetrad stage using the Trizol UP kit (TransGen Biotech, Beijing, China) and subjected to sequencing on the Illumina platform. Sequencing data quality was assessed using fastp [69] and FastQC v0.11.7 [70] and aligned to the reference genome of Zhangshugang [40] using HISAT (v2.2.1) with the default parameters [71]. Differential gene expression analysis was performed using the DESeq2 (v1.20.0) software. Genes were considered differentially expressed if they had adjusted *P*-value <0.01 and a |log_2_fold change (FC)| ≥ 2, determined using the negative binomial Wald test followed by Benjamini–Hochberg correction [72].

### Gene enrichment analysis

GO enrichment analysis categorized DEGs into biological processes (BP), cellular components (CC), and molecular functions (MF). Enrichment analysis and visualization were executed using the GOATOOLS tool [73] with a false discovery rate (FDR) threshold of <0.05.

KEGG pathway analysis was performed using KofamKOALA [74] for protein sequence homology searches. KEGG pathway enrichment analysis was carried out using R package clusterProfiler [75], with a significance threshold set at a *P*-value cutoff of 0.05.

### TRV2 virus-induced gene silencing experiments

The optimized TRV2-based VIGS system was applied in our study [76]. The targeted regions for silencing the gene of interest were selected using the VIGS tool on the Sol Genomics Network website (https://solgenomics.sgn.cornell.edu/). The TRV2:*0* (empty vector), TRV2:*PDS* (phytoene desaturase, positive control), and TRV2:*Caz06g28920* (hereafter abbreviated as TRV2:*920*) vectors were transformed into *Agrobacterium tumefaciens* strain GV3101 using the freeze–thaw method. *Agrobacterium tumefaciens* cells containing TRV1 were mixed with TRV2:*0*, TRV2:*PDS*, and TRV2:*920* vectors, respectively, in a 1:1 ratio (OD600 = 0.5). This mixture was then used to inoculate two expanded true leaves of F_1_ (9704A × Zhangshugang) seedlings using 1-ml needle-less syringes. The *A. tumefaciens*-inoculated plants were maintained in a growth chamber at 18°C in darkness for 2 days and subsequently transferred to a greenhouse with a 16 h light/8 h dark photoperiod cycle and 60% relative humidity at 22°C (day) and 18°C (night). Flower buds at the tetrad stage were collected from three randomly selected TRV2:*920*-infiltrated plants to evaluate the efficiency of *Caz06g28920* silencing.

### Creation of PepperSNP50K liquid-phase breeding chip

The liquid-phase breeding microarray data for peppers in this study were derived from resequencing 176 pepper germplasm resources provided by Hunan Agricultural University, alongside resequencing data of over 300 pepper species, including cultivars, wild species, and breeding varieties from gene banks across Asia, the Americas, Africa, and Europe. Samples were rigorously inspected for quality, and only qualified samples underwent DNA sequencing library construction. Whole-genome resequencing was performed using the MGI DNA Library Prep kit, followed by sequencing on the DNBSEQ-T7 platform (MGI Shenzhen, China) using the PE150 sequencing strategy with an average depth of 10× per sample. Each library generated ~30 Gb of sequencing data. Quality control of sequencing reads was conducted using fastp software [69], which involved removing bases with quality scores <20 for >50% of bases, sequences with >5 N base, and adapter trimming. Subsequently, variant calling was performed using the Sentieon DNAseq pipeline (v202112.06) [77]. The pipeline employed the Sentieon bwa mem function for read alignment to the reference genome, —algo LocusCollector and —algo Dedup functions for removing duplicate reads, —algo Haplotyper function for variant calls in GVCF format, and —algo GVCFtyper function for joint calling by combining GVCFs across all samples to generate a population VCF.

Candidate loci were screened based on specific criteria: SNP loci with heterozygosity <0.2, locus deletion frequency <0.1, minimal allele frequency >0.1, locus polymorphism >0.15, and sequencing depth >5×. The sequences flanking each SNP locus by 50 bp upstream and downstream were extracted and analyzed for specificity and GC content to ensure suitability for probe design. Following the principle of uniform locus distribution across the pepper chromosome, we systematically screened SNP loci. Additionally, genes related to key agronomic traits, such as disease resistance, fruit color, spicy flavor, and male sterility, as reported in the literature, were identified. High-quality SNP loci within the 1-kb intervals around these genes and their upstream and downstream regions were selected, which together constituted the PepperSNP50K liquid microarray for peppers.

### Gene BSA mapping using PepperSNP50K

DNA was extracted from the mixing pools of fertile and infertile peppers, each pool comprising more than 30 individual plants. Whole-genome fragment libraries were prepared using a modified protocol compatible with MGI’s genomic sequencing platform. In brief, 500 ng of pepper genomic DNA was sheared into fragments sized between 200 and 300 bp. Subsequently, adapters suitable for the DNBSEQ-T7 platform were added after end repair and 3′-A addition. The constructed gene fragment libraries were hybridized with probes designed on the Pepper50K liquid-phase breeding microarray. These biotinylated target-specific short fragments bound specifically to their complementary targets and were captured by streptavidin magnetic beads. The captured DNA fragments were enriched via standard PCR amplification, followed by circularization in preparation for sequencing on a DNBSEQ-T7 platform (MGI Shenzhen, China) with a PE150 module. To ensure data accuracy, each target region was sequenced to a depth of ~100×.

Raw sequencing reads were filtered using previously described criteria. Further refinement was conducted using VCF tools [78] to identify SNP loci located on chromosomes devoid of deletion or dimorphism for subsequent BSA analysis. Based on SNP genotype and sequencing depth differences across the mixed pools, allele segregation was assessed using Euclidean distance as a metric. Euclidean distance was chosen for its ability to measure segregation without requiring parental strain information and for its resilience against noise [79].

### PepperSNP50K detects the background recovery rate in backcross populations

Molecular marker-assisted backcross breeding was performed on the multi-generation backcross populations depicted in Fig. 8a using PepperSNP50K. Single, heterozygous, and missing markers were eliminated from SNP detection data, focusing on screening SNP loci. The background recovery rate was calculated as follows:

Background recovery rate = (*a* × 2 + *b*) / [(*a* + *b* + *c*) × 2] × 100%

where *a* represents the number of homozygous genotype loci identical to the donor (e.g. AA), *b* stands for the number of heterozygous genotype loci (Aa), and *c* denotes the number of homozygous genotype loci (aa) identical to the recipient.

### Double haploid technical method

The pepper materials were provided by the Pepper Research Group of Hunan Xiangyan Seed Industry. The seedlings were cultivated in a greenhouse with temperature controlled at 26–30°C during the day and 15–20°C at night. Healthy plants were selected during the flowering period, typically when the plants had bloomed to four fruits, harvested between 8 and 10.00 a.m. on sunny days. Buds with petal length equal to the sepals and in the uninucleate stage during the microspore development period were refrigerated at 4°C for 48 h. On a clean bench, the flower buds were surface-disinfected with 70% alcohol for 30 s followed by 5% sodium hypochlorite for 10–12 min and rinsed three times with sterile water. Using tweezers, anthers were carefully extracted from the buds, ensuring complete removal of filaments, and inoculated into the induction medium (NTH basic medium +0.2 mg/l NAA + 1.0 mg/l KT + 30 g/l sucrose +8 g/l agar powder). The culture dishes containing inoculated anthers were kept in the dark at 28°C until embryoids appeared. Embryoids were then transferred to the rooting medium (1/2 MS + 0.1 mg/l NAA + 20 g/l sucrose +8 g/l agar powder) for rooting. Tissue-cultured seedlings were hardened and transplanted into the substrate. After 2 weeks, 0.2% colchicine was applied to induce diploid formation. The diploid seedlings were transplanted into a field greenhouse and managed similarly to field-grown plants. Fruits were harvested upon ripening.

## Supplementary Material

Web_Material_uhae223

## Data Availability

The reference genome sequences used in this study are available at PepperGD (http://ted.bti.cornell.edu/cgi-bin/pepper/index). The raw resequencing and transcriptome sequencing data are deposited in the GSA (https://ngdc.cncb.ac.cn/gsa/) under project ID PRJCA027547 and PRJCA013331.
