# Supplementary material for: Fertility restorer gene CaRf and PepperSNP50K provide a promising breeding system for hybrid pepper
Source: Hortic Res. 2024 Oct 1;11(10):uhae223. doi: 10.1093/hr/uhae223 (PMC11480663; doi:10.1093/hr/uhae223)
Supplement: Web_Material_uhae223 [file web_material_uhae223.zip › Supplement Figures.pdf]

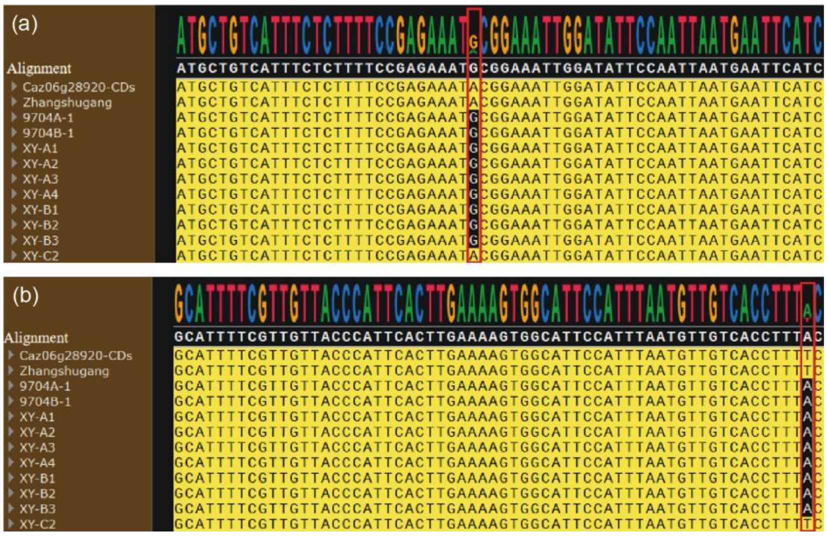

Figure S1. Comparison of missense mutation sites of *Caz06g28920* in three pepper lines. (a) At the 285th position of the CDS of the *Caz06g28920* gene, the restorer line has adenine (A), while the sterile and maintainer lines have guanine (G). (b) At the 427th position of the CDS of the *Caz06g28920* gene, the restorer line has thymine (T), while the sterile and maintainer lines have adenine (A).

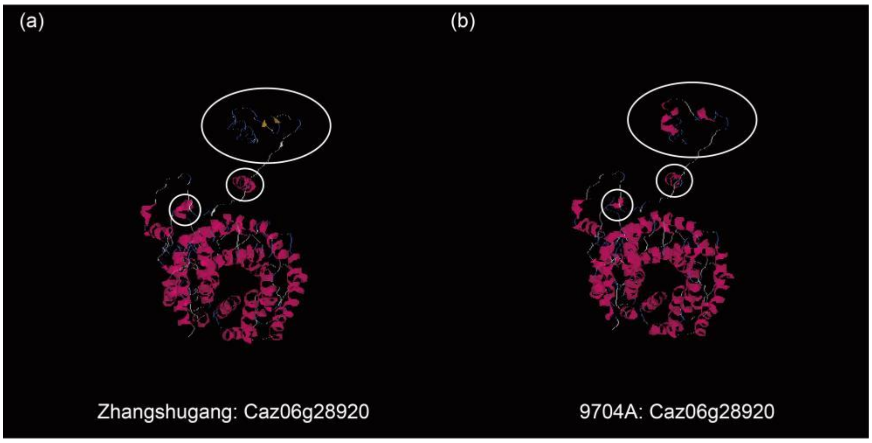

Figure S2. Tertiary structure prediction of Caz06g28920 protein in Zhangshugang (a) and 9704A (b) materials using I-TASSER prediction.

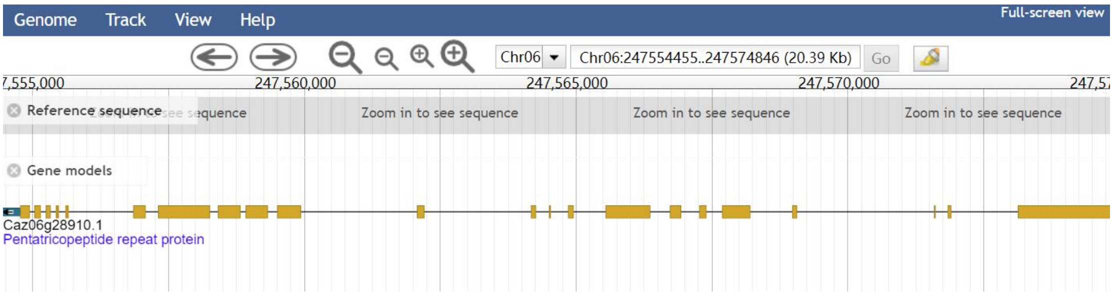

Figure S3. *Caz06g28910* gene structure.

Caz06g28910	CaT2T06g03522	89.98	1616	153	1	93	1708	72	1678
Caz06g28910	CaT2T06g03547	85.79	1696	238	1	17	1709	20	1715
Caz06g28910	CaT2T06g03583	100.00	1707	0	0	1	1707	1	1707
Caz06g28910	CaT2T06g03672	92.98	1752	114	1	1	1752	1	1743
Caz06g28910	CaT2T06g03685	96.80	1752	47	1	1	1752	1	1743
Caz06g28910	CaT2T06g03708	90.30	959	92	1	1	959	527	1484
Caz06g28910	CaT2T06g03711	92.80	1709	123	0	1	1709	1	1709

Figure S4. The CDS sequence of *Caz06g28910* gene obtained by JCVI collinearity analysis was aligned to the coding gene of CaT2T genome.

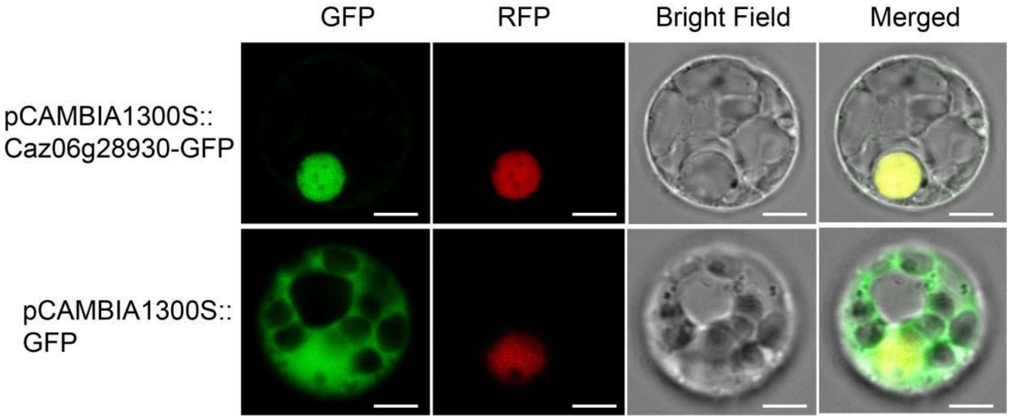

Figure S5. Subcellular localization of Caz06g28930 in rice protoplasts.

Co-localization of Caz06g28930-GFP and Ghd7-RFP in the nucleus; GFP, green fluorescent protein; Ghd7-RFP (red). Scale bars, 10 μm.

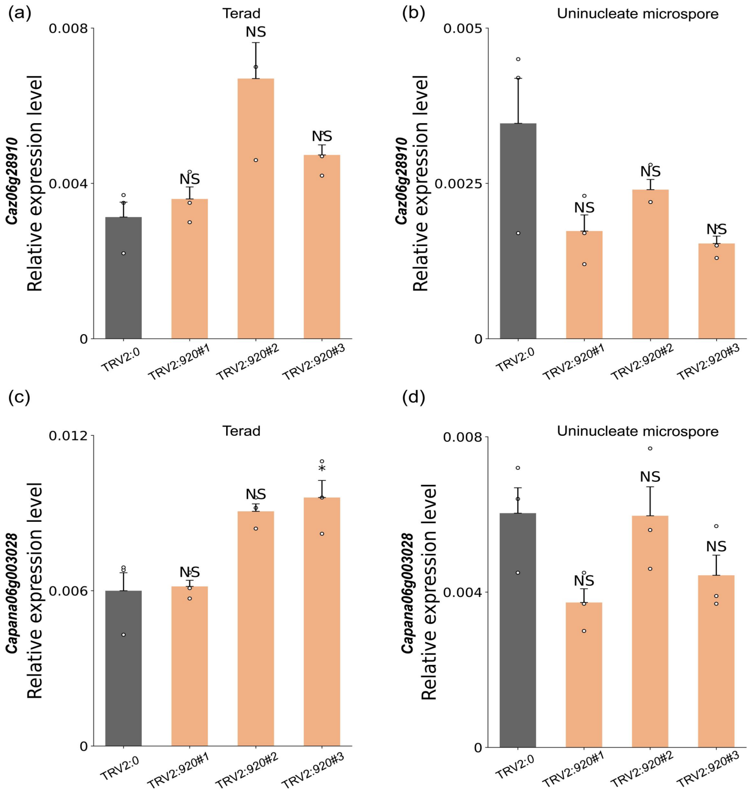

Figure S6 Expression levels of *Caz06g28910* (a, b) and *Capana06g003028* (c, d) in virus-induced

(*Caz06g28920*) F_1 (9704A × Zhangshugang) plants

Note: Gene expression was analyzed using qRT-PCR on flower buds at tetrad and uninucleate stages. Relative expression levels in TRV2:*920#1*, TRV2:*920#2,* and TRV2:*920#3* plants were compared to those in TRV2:*0*. * and ** indicate significance levels of $P < 0.05$ and $P < 0.01$, respectively, as determined by t-test. Relative expression levels are presented as mean ± SD, N = 3.

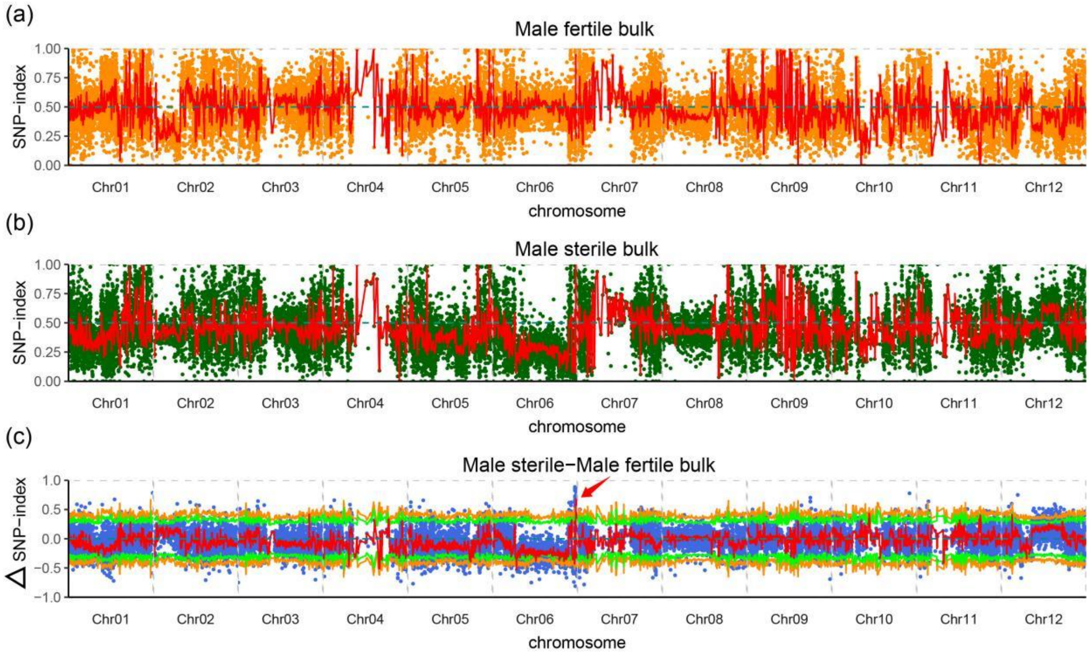

Figure S7. SNP-index distribution on chromosomes by PepperSNP50K.
