# Supplementary material for: Fertility restorer gene CaRf and PepperSNP50K provide a promising breeding system for hybrid pepper
Source: Hortic Res. 2024 Oct 1;11(10):uhae223. doi: 10.1093/hr/uhae223 (PMC11480663; doi:10.1093/hr/uhae223)
Supplement: Web_Material_uhae223 [file web_material_uhae223.zip › Supplement Tables1-9.pdf]

Table S1. Genetics analysis of male fertility and sterility in different segregated populations

Cross combination	Population	Male fertility	Male sterility	Expected ratio	χ^2	Probability
9704A×Zhangshugang	F_1	70	0	-	-	-
9704A×Zhangshugang	F_2	985	305	3:01	1.266	0.26

Table S2. Statistics of BSA mixed pool sequencing comparison results bsa

Sample ID	Genome length	Reads	GC%	Q30%	Coverage	Average Depth
L9704AP	3024945144	3008521087	35.89%	92.79%	0.9946	20.99×
LWT20AP	3024945144	3007354992	35.99%	90.06%	0.9942	18.48×
9704AP	3024945144	2998503778	35.61%	90.50%	0.9913	10.53×

Table S3. Information on KASP primers developed in this research

KASP name	Physical Location of SNPs	Primer sequence
M2403	240306419	GAAGGTGACCAAGTTCATGCTGATAGGTCTGCGTGCACAGC
		GAAGGTCGGAGTCAACGGATTGATAGGTCTGCGTGCACAGA
		AACGACATACCCAGTGTAATCACA
M2460	246009902	GAAGGTGACCAAGTTCATGCTCGTCATGTTGCCCTGCCTA
		GAAGGTCGGAGTCAACGGATTCGTCATGTTGCCCTGCCTG
		AAAGCTGAGGGAATCTAAGGTGTT
M2465	246564243	GAAGGTGACCAAGTTCATGCTGTAAAACTCAAAGCAGGGAGAAC
		GAAGGTCGGAGTCAACGGATTGTAAAACTCAAAGCAGGGAGAAA
		CACCCCAAAGTTTCAAAGTCA
M2467	246736279	GAAGGTGACCAAGTTCATGCTAGAAACAAGGTAATTTACAGAGAAAGAAA
		GAAGGTCGGAGTCAACGGATTAGAAACAAGGTAATTTACAGAGAAAGAAG
		TGGGCATCTTGTGTGTAGTTATCA
M2469	246954384	GAAGGTGACCAAGTTCATGCTTTGAAGAGATCAAGGAGGACG
		GAAGGTCGGAGTCAACGGATTTTGAAGAGATCAAGGAGGACA
		TTGTCGTTTAGCAGGACTTGA
M2473	247327150	GAAGGTGACCAAGTTCATGCTCAACAGTGGGGCAACCAATAC
		GAAGGTCGGAGTCAACGGATTCAACAGTGGGGCAACCAATAT
		GTTTGAGTTGGAGGCCATTT
M2476	247646884	GAAGGTGACCAAGTTCATGCTAGTTGAAGTAGACACCACTTGG
		GAAGGTCGGAGTCAACGGATTAGTTGAAGTAGACACCACTTGT
		ACGGTGAAAGTGATGACTGGA
M2477	247760288	GAAGGTGACCAAGTTCATGCTGAGTAGGAGCTACAGCAACTGC
		GAAGGTCGGAGTCAACGGATTGAGTAGGAGCTACAGCAACTGT

		GTGCATTTATGCTTTGGGATG
M2482	248221876	GAAGGTGACCAAGTTCATGCTCCCCGATCAATTTTCTAAGCAC
		GAAGGTCGGAGTCAACGGATTCCCCGATCAATTTTCTAAGCAA
		TGTGGCTATTTCGGTGGACTA
M2486	248640749	GAAGGTGACCAAGTTCATGCTTCTCAAGAGCCCTCTAGCTCC
		GAAGGTCGGAGTCAACGGATTTCTCAAGAGCCCTCTAGCTCG
		TGATTGGACGTGGAGAGGTAA
M2475	247518836	GAAGGTGACCAAGTTCATGCTCAAGCTATATTTGGTATTGGTGTGC
		GAAGGTCGGAGTCAACGGATTCAAGCTATATTTGGTATTGGTGTGT
		TGTCATTCAGTGATTTGTCCGTTT
M2489	248955305	GAAGGTGACCAAGTTCATGCTCATATGAACCCCATACATTCAGCC
		GAAGGTCGGAGTCAACGGATTCATATGAACCCCATACATTCAGCG
		AGCTCGTGTCTCTGAACCTATTTT
M2495	249540756	GAAGGTGACCAAGTTCATGCTTTGACGTTATTCATGGACTAC
		GAAGGTCGGAGTCAACGGATTTTGACGTTATTCATGGACTAT
		TCACCATGTTCTCATCTGCTAAA
M2499	249998056	GAAGGTGACCAAGTTCATGCTCGTATTAAGGGAATACAAGACCTA
		GAAGGTCGGAGTCAACGGATTCGTATTAAGGGAATACAAGACCTG
		GCTCAATGTGGCATAGCTCA
M2507	250709232	GAAGGTGACCAAGTTCATGCTCCTCACTCTCCAATTGTATGATCAATAT
		GAAGGTCGGAGTCAACGGATTCCTCACTCTCCAATTGTATGATCAATAG
		TATTCATGCTTCCTTCCGTAAGCT
M2530	253004872	GAAGGTGACCAAGTTCATGCTGGACCGAAAATCAACCTGGGTAC

Table S4. F_2 population important recombinant single plant

Serial number	Fertility	Marker genotype							
		M2465	M2467	M2469	M2473	M2475	M2476	M2477	M2482
Zhangshugang	MF	*RfRf*	*RfRf*	*RfRf*	*RfRf*	*RfRf*	*RfRf*	*RfRf*	*RfRf*
9704A	MS	*rfrf*	*rfrf*	*rfrf*	*rfrf*	*rfrf*	*rfrf*	*rfrf*	*rfrf*
F2-144	MF	*rfrf*	*rfrf*	*rfrf*	*Rfrf*	*Rfrf*	*rfrf*	*rfrf*	*rfrf*
F2-241	MF	*rfrf*	*rfrf*	*Rfrf*	*Rfrf*	*Rfrf*	*Rfrf*	*Rfrf*	*Rfrf*
F2-472	MF	*rfrf*	*rfrf*	*rfrf*	*Rfrf*	*Rfrf*	*rfrf*	*rfrf*	*rfrf*
F2-478	MF	*Rfrf*	*Rfrf*	*Rfrf*	*Rfrf*	*Rfrf*	*Rfrf*	*Rfrf*	*rfrf*
F2-909	MF	*rfrf*	*Rfrf*	*Rfrf*	*Rfrf*	*Rfrf*	*Rfrf*	*Rfrf*	*Rfrf*
F2-1218	MF	*Rfrf*	*Rfrf*	*Rfrf*	*Rfrf*	*Rfrf*	*Rfrf*	*Rfrf*	*rfrf*
F2-1350	MF	*Rfrf*	*Rfrf*	*Rfrf*	*Rfrf*	*Rfrf*	*Rfrf*	*Rfrf*	*rfrf*
F2-37	MS	*Rfrf*	*rfrf*	*rfrf*	*rfrf*	*rfrf*	*rfrf*	*rfrf*	*rfrf*
F2-175	MS	*rfrf*	*rfrf*	*rfrf*	*rfrf*	*rfrf*	*rfrf*	*rfrf*	*Rfrf*
F2-207	MS	*rfrf*	*rfrf*	*rfrf*	*rfrf*	*rfrf*	*rfrf*	*rfrf*	*Rfrf*
F2-231	MS	*rfrf*	*rfrf*	*rfrf*	*rfrf*	*rfrf*	*rfrf*	*rfrf*	*Rfrf*
F2-232	MS	*rfrf*	*rfrf*	*rfrf*	*rfrf*	*rfrf*	*rfrf*	*rfrf*	*Rfrf*
F2-893	MS	*rfrf*	*rfrf*	*rfrf*	*rfrf*	*rfrf*	*rfrf*	*rfrf*	*RfRf*
F2-905	MS	*rfrf*	*rfrf*	*rfrf*	*rfrf*	*rfrf*	*rfrf*	*rfrf*	*RfRf*
F2-917	MS	*rfrf*	*rfrf*	*rfrf*	*rfrf*	*rfrf*	*rfrf*	*rfrf*	*RfRf*

F2-931	MS	*rfrf*	*rfrf*	*rfrf*	*rfrf*	*rfrf*	*rfrf*	*rfrf*	*RfRf*
F2-935	MS	*rfrf*	*rfrf*	*rfrf*	*rfrf*	*rfrf*	*rfrf*	*rfrf*	*RfRf*
F2-944	MS	*rfrf*	*rfrf*	*rfrf*	*rfrf*	*rfrf*	*rfrf*	*rfrf*	*RfRf*
F2-947	MS	*Rfrf*	*rfrf*	*rfrf*	*rfrf*	*rfrf*	*rfrf*	*rfrf*	*rfrf*
F2-949	MS	*rfrf*	*rfrf*	*rfrf*	*rfrf*	*rfrf*	*rfrf*	*rfrf*	*RfRf*
F2-957	MS	*rfrf*	*rfrf*	*rfrf*	*rfrf*	*rfrf*	*rfrf*	*rfrf*	*RfRf*
F2-960	MS	*rfrf*	*rfrf*	*rfrf*	*rfrf*	*rfrf*	*rfrf*	*rfrf*	*RfRf*
F2-965	MS	*rfrf*	*rfrf*	*rfrf*	*rfrf*	*rfrf*	*rfrf*	*rfrf*	*RfRf*
F2-998	MS	*rfrf*	*rfrf*	*rfrf*	*rfrf*	*rfrf*	*rfrf*	*rfrf*	*RfRf*
F2-1000	MS	*rfrf*	*rfrf*	*rfrf*	*rfrf*	*rfrf*	*rfrf*	*rfrf*	*RfRf*
F2-1006	MS	*rfrf*	*rfrf*	*rfrf*	*rfrf*	*rfrf*	*rfrf*	*rfrf*	*RfRf*
F2-1012	MS	*rfrf*	*rfrf*	*rfrf*	*rfrf*	*rfrf*	*rfrf*	*rfrf*	*RfRf*
F2-1032	MS	*rfrf*	*rfrf*	*rfrf*	*rfrf*	*rfrf*	*rfrf*	*rfrf*	*RfRf*
F2-1040	MS	*rfrf*	*rfrf*	*rfrf*	*rfrf*	*rfrf*	*rfrf*	*rfrf*	*RfRf*
F2-1045	MS	*rfrf*	*rfrf*	*rfrf*	*rfrf*	*rfrf*	*rfrf*	*rfrf*	*RfRf*
F2-1049	MS	*rfrf*	*rfrf*	*rfrf*	*rfrf*	*rfrf*	*rfrf*	*rfrf*	*RfRf*
F2-1064	MS	*rfrf*	*rfrf*	*rfrf*	*rfrf*	*rfrf*	*rfrf*	*rfrf*	*RfRf*
F2-1065	MS	*rfrf*	*rfrf*	*rfrf*	*rfrf*	*rfrf*	*rfrf*	*rfrf*	*RfRf*
F2-1081	MS	*rfrf*	*rfrf*	*rfrf*	*rfrf*	*rfrf*	*rfrf*	*rfrf*	*RfRf*
F2-1096	MS	*rfrf*	*rfrf*	*rfrf*	*rfrf*	*rfrf*	*rfrf*	*rfrf*	*RfRf*
F2-1099	MS	*rfrf*	*rfrf*	*rfrf*	*rfrf*	*rfrf*	*rfrf*	*rfrf*	*RfRf*
F2-1103	MS	*rfrf*	*rfrf*	*rfrf*	*rfrf*	*rfrf*	*rfrf*	*rfrf*	*RfRf*
F2-1104	MS	*rfrf*	*rfrf*	*rfrf*	*rfrf*	*rfrf*	*rfrf*	*rfrf*	*RfRf*
F2-1116	MS	*rfrf*	*rfrf*	*rfrf*	*rfrf*	*rfrf*	*rfrf*	*rfrf*	*RfRf*
F2-1127	MS	*rfrf*	*rfrf*	*rfrf*	*rfrf*	*rfrf*	*rfrf*	*rfrf*	*RfRf*
F2-1132	MS	*rfrf*	*rfrf*	*rfrf*	*rfrf*	*rfrf*	*rfrf*	*rfrf*	*RfRf*
F2-1137	MS	*rfrf*	*rfrf*	*rfrf*	*rfrf*	*rfrf*	*rfrf*	*rfrf*	*RfRf*
F2-1143	MS	*rfrf*	*rfrf*	*rfrf*	*rfrf*	*rfrf*	*rfrf*	*rfrf*	*RfRf*
F2-1146	MS	*rfrf*	*rfrf*	*rfrf*	*rfrf*	*rfrf*	*rfrf*	*rfrf*	*RfRf*
F2-1159	MS	*rfrf*	*rfrf*	*rfrf*	*rfrf*	*rfrf*	*rfrf*	*rfrf*	*RfRf*
F2-1160	MS	*rfrf*	*rfrf*	*rfrf*	*rfrf*	*rfrf*	*rfrf*	*rfrf*	*RfRf*
F2-1162	MS	*rfrf*	*rfrf*	*rfrf*	*rfrf*	*rfrf*	*rfrf*	*rfrf*	*RfRf*
F2-1172	MS	*rfrf*	*rfrf*	*rfrf*	*rfrf*	*rfrf*	*rfrf*	*rfrf*	*RfRf*
F2-1192	MS	*rfrf*	*rfrf*	*rfrf*	*rfrf*	*rfrf*	*rfrf*	*rfrf*	*RfRf*
F2-1195	MS	*rfrf*	*rfrf*	*rfrf*	*rfrf*	*rfrf*	*rfrf*	*rfrf*	*RfRf*
F2-1205	MS	*rfrf*	*rfrf*	*rfrf*	*rfrf*	*rfrf*	*rfrf*	*rfrf*	*RfRf*
F2-1217	MS	*rfrf*	*rfrf*	*rfrf*	*rfrf*	*rfrf*	*rfrf*	*rfrf*	*RfRf*
F2-1232	MS	*rfrf*	*rfrf*	*rfrf*	*rfrf*	*rfrf*	*rfrf*	*rfrf*	*RfRf*
F2-1237	MS	*rfrf*	*rfrf*	*rfrf*	*rfrf*	*rfrf*	*rfrf*	*rfrf*	*RfRf*
F2-1241	MS	*rfrf*	*rfrf*	*rfrf*	*rfrf*	*rfrf*	*rfrf*	*rfrf*	*RfRf*
F2-1274	MS	*rfrf*	*rfrf*	*rfrf*	*rfrf*	*rfrf*	*rfrf*	*rfrf*	*RfRf*
F2-1277	MS	*rfrf*	*rfrf*	*rfrf*	*rfrf*	*rfrf*	*rfrf*	*rfrf*	*RfRf*
F2-1286	MS	*rfrf*	*rfrf*	*rfrf*	*rfrf*	*rfrf*	*rfrf*	*rfrf*	*RfRf*
F2-1305	MS	*rfrf*	*rfrf*	*rfrf*	*rfrf*	*rfrf*	*rfrf*	*rfrf*	*Rfrf*

F2-1313	MS	*rfrf*	*rfrf*	*rfrf*	*rfrf*	*rfrf*	*rfrf*	*rfrf*	*RfRf*
F2-1320	MS	*rfrf*	*rfrf*	*rfrf*	*rfrf*	*rfrf*	*rfrf*	*rfrf*	*RfRf*
F2-1328	MS	*rfrf*	*rfrf*	*rfrf*	*rfrf*	*rfrf*	*rfrf*	*rfrf*	*RfRf*
F2-1331	MS	*rfrf*	*rfrf*	*rfrf*	*rfrf*	*rfrf*	*rfrf*	*rfrf*	*RfRf*
F2-1334	MS	*rfrf*	*rfrf*	*rfrf*	*rfrf*	*rfrf*	*rfrf*	*rfrf*	*RfRf*
F2-1337	MS	*rfrf*	*rfrf*	*rfrf*	*rfrf*	*rfrf*	*rfrf*	*rfrf*	*RfRf*
F2-1339	MS	*rfrf*	*rfrf*	*rfrf*	*rfrf*	*rfrf*	*rfrf*	*rfrf*	*RfRf*
F2-1341	MS	*rfrf*	*rfrf*	*rfrf*	*rfrf*	*rfrf*	*rfrf*	*rfrf*	*RfRf*
F2-1343	MS	*rfrf*	*rfrf*	*rfrf*	*rfrf*	*rfrf*	*rfrf*	*rfrf*	*RfRf*
F2-1345	MS	*rfrf*	*rfrf*	*rfrf*	*rfrf*	*rfrf*	*rfrf*	*rfrf*	*RfRf*
F2-1346	MS	*rfrf*	*rfrf*	*rfrf*	*rfrf*	*rfrf*	*rfrf*	*rfrf*	*RfRf*
F2-1349	MS	*rfrf*	*rfrf*	*rfrf*	*rfrf*	*rfrf*	*rfrf*	*rfrf*	*RfRf*
F2-1353	MS	*rfrf*	*rfrf*	*rfrf*	*rfrf*	*rfrf*	*rfrf*	*rfrf*	*RfRf*

Note: MF represents fertile phenotype and MS represents sterile phenotype.

Table S5 The genotype of the *Rf*-related PARMS markers in *Capsicum annuum* L. accessions.

Accessions	Phenotype	Marker genotype						
		M2465	M2469	M2473	M2475	M2476	M2477	M2482
XY-A1	CMS line	*rfrf*	*rfrf*	*rfrf*	*rfrf*	*rfrf*	*rfrf*	*RfRf*
XY-A2	CMS line	*rfrf*	*rfrf*	*rfrf*	*rfrf*	*rfrf*	*rfrf*	*RfRf*
XY-A3	CMS line	*rfrf*	*rfrf*	*rfrf*	*rfrf*	*rfrf*	*rfrf*	*RfRf*
XY-A4	CMS line	*rfrf*	*rfrf*	*rfrf*	*rfrf*	*rfrf*	*rfrf*	*RfRf*
XY-A5	CMS line	*rfrf*	*rfrf*	*rfrf*	*rfrf*	*rfrf*	*rfrf*	*RfRf*
XY-C1	Restorer line	*rfrf*	*RfRf*	*RfRf*	*RfRf*	*RfRf*	*RfRf*	*RfRf*
XY-C2	Restorer line	*rfrf*	*RfRf*	*RfRf*	*RfRf*	*rfrf*	*rfrf*	*RfRf*
XY-C3	Restorer line	*rfrf*	*RfRf*	*RfRf*	*RfRf*	*rfrf*	*rfrf*	*RfRf*
XY-C4	Restorer line	*rfrf*	*RfRf*	*RfRf*	*RfRf*	*rfrf*	*rfrf*	*RfRf*
XY-C5	Restorer line	*rfrf*	*rfrf*	*RfRf*	*RfRf*	*rfrf*	*rfrf*	*RfRf*
XY-B1	Maintainer line	*rfrf*	*rfrf*	*rfrf*	*rfrf*	*rfrf*	*rfrf*	*RfRf*
XY-B2	Maintainer line	*rfrf*	*rfrf*	*rfrf*	*rfrf*	*rfrf*	*rfrf*	*RfRf*
XY-B3	Maintainer line	*rfrf*	*rfrf*	*rfrf*	*rfrf*	*RfRf*	*RfRf*	*RfRf*
Rate of successful genotyping (%)		61.54%	92.31%	100%	100%	61.54%	61.54%	38.46%

Table S6. Gene annotation information in *CaRf* candidate interval

Gene ID	Pos	Annotation
Caz06g28830	246950820	TPR_REGION domain-containing protein
Caz06g28840	247204413	NAC domain-containing protein
Caz06g28850	247230122	NAC domain-containing protein
Caz06g28860	247247740	DUF4283 domain-containing protein
Caz06g28870	247258311	NAC domain-containing protein
Caz06g28880	247471302	NB-ARC domain-containing protein
Caz06g28890	247507321	NB-ARC domain-containing protein
Caz06g28900	247550745	protein TRANSPORT INHIBITOR RESPONSE 1

Caz06g28910	247554451	Pentatricopeptide repeat protein
Caz06g28920	247585338	Pentatricopeptide repeat protein
Caz06g28930	247614078	Pentatricopeptide repeat protein
Caz06g28940	247616398	Protein TRANSPORT INHIBITOR RESPONSE 1

Table S7. Chromosomes distribution and basic information of loci

Chromosome	Chromosome length	SNP counts	SNP start	SNP end	Maximum spacing	Average distance (bp)	Coverage
Chr01	332615375	5739	37090	332169520	669821	57957	99.85%
Chr02	177319215	3041	119269	177226551	829088	58310	99.88%
Chr03	289790774	5010	8645	289243033	620666	57842	99.81%
Chr04	248932513	4137	279249	247082944	583929	60172	99.14%
Chr05	254874144	4217	861544	252810606	1212655	60440	98.85%
Chr06	253233553	4130	152074	252095238	796888	61316	99.49%
Chr07	266382521	4438	426352	266015184	1017868	60023	99.70%
Chr08	174326481	2864	541011	173990261	713000	60868	99.50%
Chr09	278410012	4838	22780	277494433	1202242	57547	99.66%
Chr10	210332287	3600	14446	209770205	1948984	58426	99.73%
Chr11	275185330	5002	38440	274993042	1663365	55015	99.92%
Chr12	259736375	4156	1060370	259541828	979668	62497	99.52%

Table S8. Polymorphism Summary of PepperSNP50

Quality Index	Mean
Miss rate	0.005
Heter rate	0.012
MAF	0.285
PIC	0.305
Depth	10.703
Coverage	99.59%
Distance (bp)	59201

Table S9. Other Primers used in this research

Name	Forward primer (5'-3')	Reverse primer (5'-3')
**Gene clone**		

Caz06g28920	ATGACAAGAGTTCTTGTGAGC	AGCTCTGGTATCATGTCAAGG
Caz06g28930	AAGGCATTCCTCCGGACATA	AGCTCTGGTATCATGTCAAGG
Caz06g28910-pro	ATGGCTTTCAGTCTGCCCTA	ATGGCTTTCAGTCTGCCCTA
Caz06g28920-pro	TCAATATAATTTCGAGAGTCATCCA	GCTTTCAGTCTGCCCTAATTTC
Caz06g28930-pro	GCCAATCGGACATCTTTCCATAC	GCCAATCGGACATCTTTCCATAC
**Real-time quantitative RT-PCR analysis**		
Caz06g28920	AGGGAACACTAAGCCCAACA	ATGTCCGGAGGAATGCCTTT
Caz06g28910	CCGTCATGAATGGACTCAGC	TCGGAGGAATGCCTTTCTGT
Capana06g003028	TCCAACTGTGCTCACCTTCA	ATCCACTTGACCACGCAAAC
UBI-3	TGTCCATCTGCTCTCTGTTG	CACCCCAAGCACAATAAGAC
**Subcellular localization**		
Caz06g28920-GFP	CAAATCGACTCTAGAAAGCTTATGAC AAGAGTTCTTGTGAGCA	GCCCTTGCTCACCATGGTACCAGCTCTG GTATCATGTCAAGGA
Caz06g28930-GFP	CAAATCGACTCTAGAAAGCTTATGCCT TCTGCAAAGATAGAAAAC	GCCCTTGCTCACCATGGTACCTGAAGCT CTGGTATCATGTCAA
**VIGS**		
C2b-CaPDS	TGAGGAGAAGAGCCCAGGTCTTCTTTG GGAACTGATAG	GTCGACGACAAGACCCAGACAAACCAC CCAAACCTGC
C2b-Caz06g28920	TGAGGAGAAGAGCCCCAAGAGTTCTT GTGAGCAAATCTAA	GTCGACGACAAGACCCAATATCCAATTT CCGTATTTCTCGG
